# Supplementary material for: How cells tame noise while maintaining ultrasensitive transcriptional responses
Source: PLoS Comput Biol. 2025 Dec 11;21(12):e1013217. doi: 10.1371/journal.pcbi.1013217 (PMC12721554; doi:10.1371/journal.pcbi.1013217)
Supplement: S1 Text — (DOCX) [file pcbi.1013217.s001.docx]

**S1 Text** for How Cells Tame Noise While Maintaining Ultrasensitive Transcriptional Responses

Eui Min Jeong^1☯*^, Chang Yoon Chung^1☯^, and Jae Kyoung Kim^1,2,3,4*^

**1** Biomedical Mathematics Group, Pioneer Research Center for Mathematical and Computational Sciences, Institute for Basic Science, Daejeon, Republic of Korea

**2** Department of Mathematical Sciences, KAIST, Daejeon, Republic of Korea

**3** Department of Medicine, College of Medicine, Korea University, Seoul, Republic of Korea

**4** Graduate School of Data Science, KAIST, Daejeon, Republic of Korea

☯These authors contributed equally to this work.

^*^ wjddmlals11@inha.ac.kr, jaekkim@kaist.ac.kr

**Supplementary Methods**

**Derivation of the equations for the transcriptional activity and the Fano factor of the four binding sites model with cooperative binding**

To derive equations for the transcriptional activity and the Fano factor of the four binding sites model with cooperative binding, we followed the methodology introduced by Sanchez et al [31]. Specifically, we described the transcription regulated by cooperative binding with four binding sites on DNA with the following chemical master equations (CMEs):

|  | $\frac{dp_{m}}{dt}=Kp_{m}+\beta\left( m+1 \right)p_{m+1}-\beta mp_{m}-Pp_{m}+Pp_{m-1}$, | (S1) |
| --- | --- | --- |

where

$p_{m}=\left[ p_{m}^{0000},p_{m}^{1000},p_{m}^{0100},p_{m}^{0010},p_{m}^{0001},p_{m}^{1100},p_{m}^{1010},p_{m}^{1001},p_{m}^{0110},p_{m}^{0101},p_{m}^{0011},p_{m}^{1110},p_{m}^{1101},p_{m}^{1011},p_{m}^{0111},p_{m}^{1111} \right]^{T}$

denotes the probability vector, where each component $p_{m}^{X}$ represents the joint probability of the DNA being in the state $E_{X}$ with $m$ mRNA molecules. Each state $X\in\left\{ a_{1}a_{2}a_{3}a_{4}|a_{i}\in\left\{ 0,1 \right\},i=1,2,3,4 \right\}$ encodes the occupancy of the *i*-th DNA binding site (1 if occupied, 0 otherwise). Additionally, the matrix $K=\left( k_{ij} \right)$ governs stochastic transitions between DNA states: each off-diagonal element $k_{ij}$ represents the transition rate from state *j* to state *i* (Table 1), while each diagonal element $k_{ii}$ is set to the negative sum of the other elements in its column (i.e., $k_{ii}=\sum_{j\neq i} k_{ji}$), representing the total rate of escape from state *i*. The diagonal matrix $P=diag\left( \left[ \alpha,\cdots,\alpha,0 \right] \right)$ represents the transcription rate associated with each DNA state. Note that all diagonal elements of $P$ are $\alpha$, except for the last entry corresponding to the repressed state $E_{1111}$, which is set to zero (Fig 1a).

From Eq. (S1), we can calculate the $i$-th moment vector of mRNAs at the steady state, defined as $m_{i}=\sum_{m=0}^{\infty} m^{i}p_{m}$, where each component of $m_{i}$ represents the $i$-th moment of the stationary mRNA distribution for each DNA state. For example, the zeroth moment vector $m_{0}$ is obtained by summing $p_{m}$ of Eq. (S1) over $m$ from 0 to infinity as follows:

|  | $\frac{dm_{0}}{dt}=Km_{0}+\beta\sum_{m=0}^{\infty} \left( m+1 \right)p_{m+1}-\beta\sum_{m=0}^{\infty} mp_{m}-Pm_{0}+Pm_{0}=Km_{0}.$ | (S2) |
| --- | --- | --- |

Hence, at the steady state, the zeroth moment vector satisfies $Km_{0}=0$. Thus, the transcriptional activity (denoted as $TA\left( R_{T} \right)$) defined as the probability that the transcription is active can be derived by subtracting the last element of $m_{0}$ corresponding to the repressed state $E_{1111}$ from 1 (Fig 1b):

|  | $TA\left( R_{T};c,K_{r} \right)=1-\frac{R_{T}^{4}}{c^{6}\Omega^{4}K_{r}^{4}+4c^{6}\Omega^{3}K_{r}^{3}R_{T}+6c^{5}\Omega^{2}K_{r}^{2}R_{T}^{2}+4c^{3}\Omega K_{r}R_{T}^{3}+R_{T}^{4}} .$ | (S3) |
| --- | --- | --- |

Similarly, the first and second moments vectors $m_{1}$ and $m_{2}$ can be derived by multiplying both sides of Eq. (S1) by $m$ and $m^{2}$, respectively, and summing over all $m$:

|  | $0=\left( K-\beta I \right)m_{1}+Pm_{0}$,  $0=\left( K-2\beta I \right)m_{2}+\left( 2P+\beta I \right)m_{1}+Pm_{0}$, | (S4)  (S5) |
| --- | --- | --- |

where $I$ is the identity matrix. As the total sum of elements in $m_{1}$ and $m_{2}$ are the first moment and second moment of mRNAs, $\left\langle m \right\rangle$ and $\left\langle m^{2} \right\rangle$, respectively, we can derive equations for them by multiplying both Eq. (S4) and Eq. (S5) from the left by the all-ones vector $u=\left[ \begin{matrix} 1 & 1 & \cdots& 1 \end{matrix} \right]$, as follows:

|  | $0=-\beta\left\langle m \right\rangle+u\cdot Pm_{0}$,  $0=-2\beta\left\langle m^{2} \right\rangle+2u\cdot Pm_{1}+\beta\left\langle m \right\rangle+u\cdot Pm_{0}.$ |  |
| --- | --- | --- |

Therefore, we get the following equations:

|  | $\left\langle m \right\rangle=\frac{u\cdot Pm_{0}}{\beta},$ | (S6) |
| --- | --- | --- |

and

|  | $\left\langle m^{2} \right\rangle=\frac{u\cdot Pm_{1}}{\beta}+\frac{\left\langle m \right\rangle}{2}+\frac{u\cdot Pm_{0}}{2\beta}=\frac{u\cdot Pm_{1}}{\beta}+\left\langle m \right\rangle.$ | (S7) |
| --- | --- | --- |

Combining Eq. (S6) and Eq. (S7), we can get the equation for the Fano factor, $FF\left( R_{T} \right)$, defined as the ratio of the variance to the mean of the mRNA copy number (Fig 1c):

|  | $FF\left( R_{T};c,K_{r},k_{f}\Omega^{-1} \right)=\frac{\left\langle m^{2} \right\rangle-\left\langle m \right\rangle^{2}}{\left\langle m \right\rangle} .$ | (S8) |
| --- | --- | --- |

The full derivation of the transcriptional activity and the Fano factor in the four binding site model is available in https://github.com/Mathbiomed/Ultrasensitive-gene-switch
