# Supplementary figures and images for: How cells tame noise while maintaining ultrasensitive transcriptional responses
Source: PLoS Comput Biol. 2025 Dec 11;21(12):e1013217. doi: 10.1371/journal.pcbi.1013217 (PMC12721554; doi:10.1371/journal.pcbi.1013217)

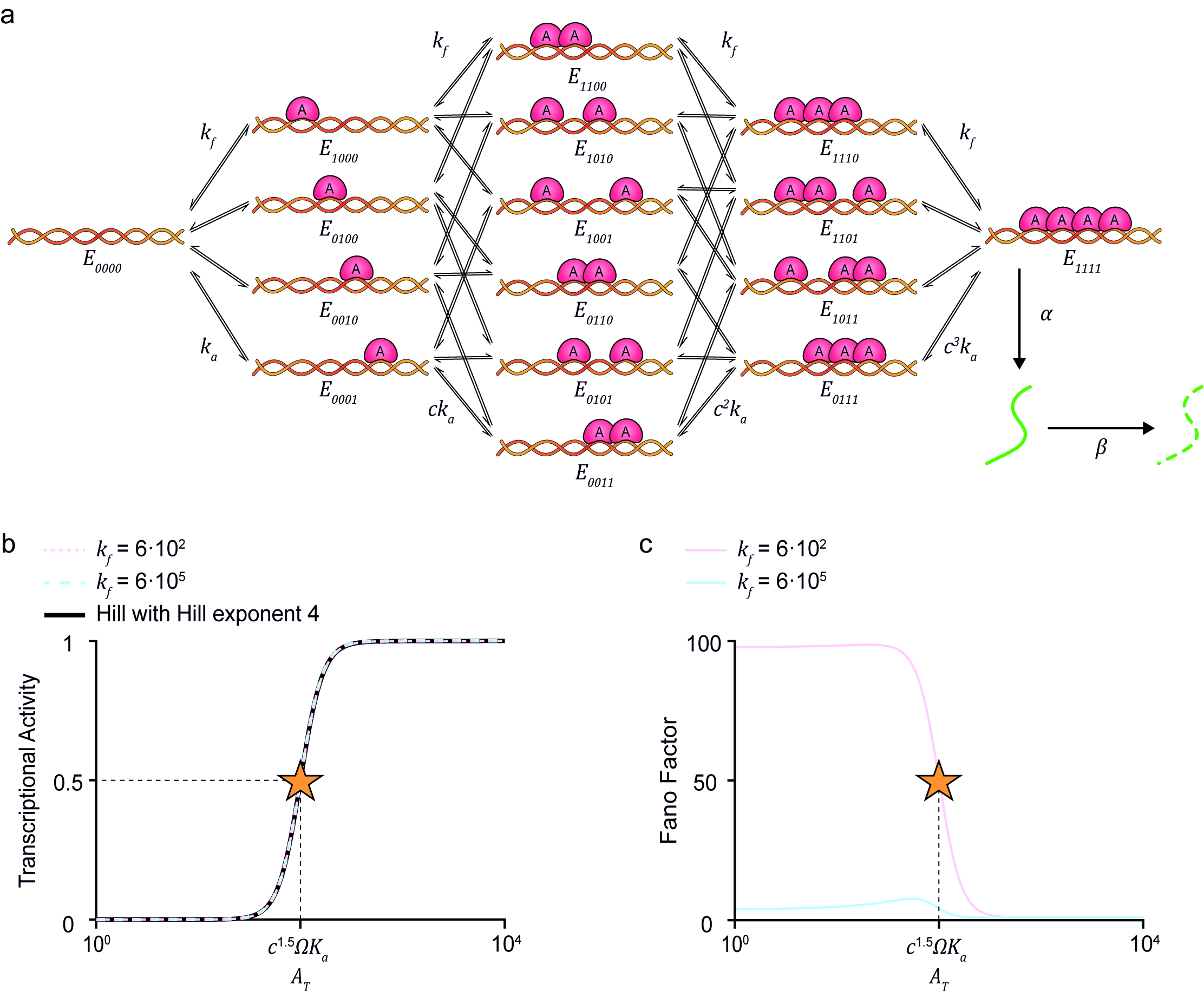

Supplement: S1 Fig — (a) Model diagram of the transcription regulated by the activator proteins (A) binding to four independent sites on the DNA within a cell volume of Ω. Each binding site is occupied by A at a rate kf. Conversely, A unbinds from DNA at a rate ka when one site is occupied, with the dissociation constant between A and DNA defined as Ka=kakf. For two, three, or four occupied sites, A unbinds at rates cka, c2ka and c3ka, respectively. Accordingly, when c<1, cooperative binding is present. When all binding sites are occupied, mRNA is produced at a rate α and degrades at a rate β, whereas transcription is inactive if any site remains unoccupied. (b) When c=10−2, transcriptional activity closely resembles the Hill function with a Hill exponent of 4 (black line). Furthermore, transcriptional activity remains consistent with the Hill function regardless of kf values, provided Ka is kept constant (red dotted line and blue dashed line). (c) Nevertheless, the overall noise level quantified by the Fano factor of mRNAs shows significant differences based on kf. Notably, the overall noise levels are reduced during the sensitive response when kf is faster (red solid line) compared to when it is slower (blue solid line). (TIF) [file pcbi.1013217.s002.tif]

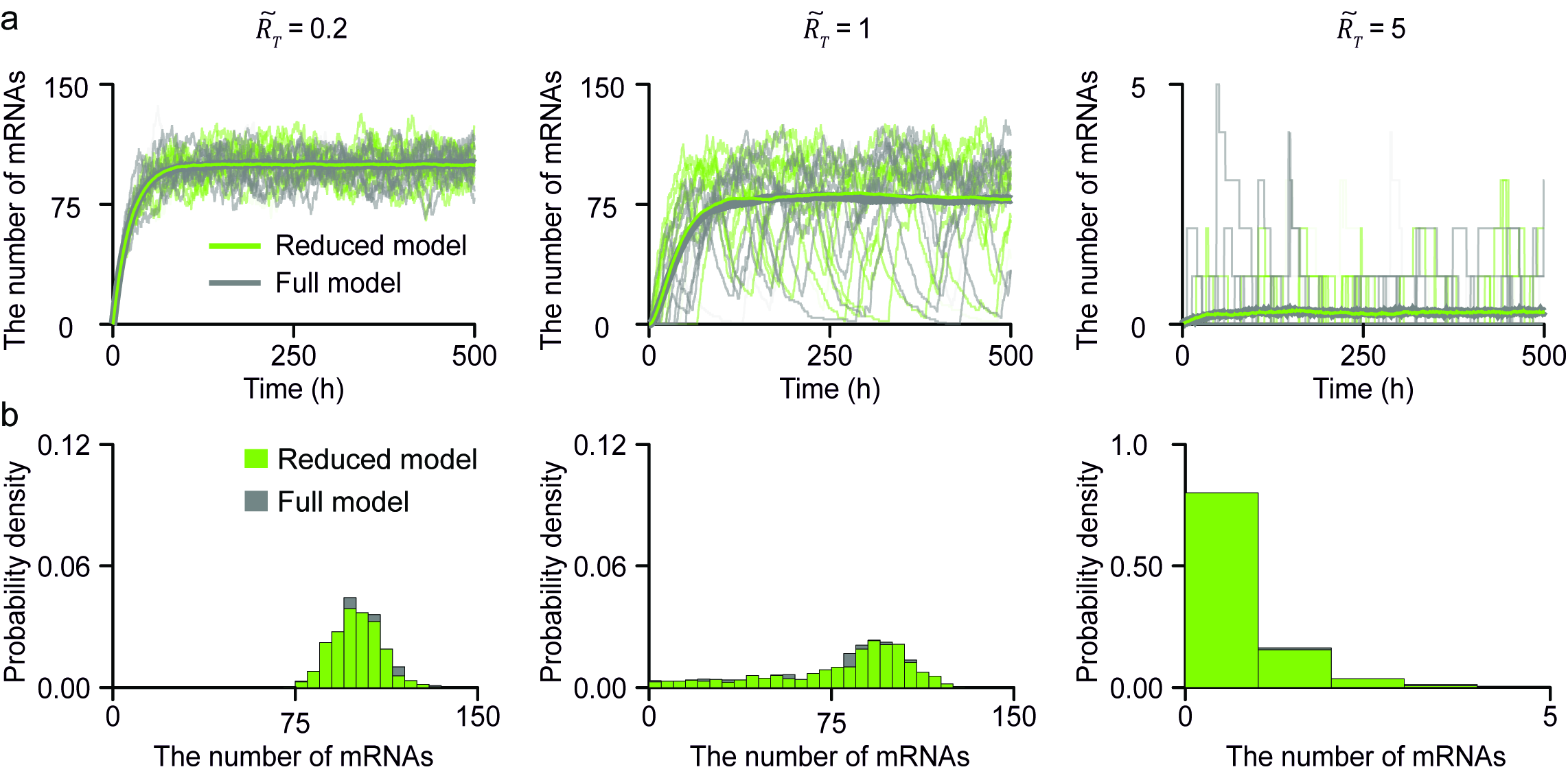

Supplement: S2 Fig — (a) Ten representative time-series of mRNA copy numbers (thin lines) and their mean trajectories (thick lines) were obtained from 1,000 stochastic simulations using the model that combines sequestration, blocking, and displacement. The reduced model, in which the numbers of activators and repressors are approximated by their QSSA (gray lines; see Methods), was compared with the full model that explicitly models the binding and unbinding reactions between them (green lines). Simulations were performed while varying the molar ratio RT~=0.2 (left), 1 (middle), and 5 (right), with the original parameter set in Table 1. (b) Probability density functions of the simulated mRNA copy numbers at 500 h, obtained from 1,000 stochastic simulation runs varying the molar ratio RT~=0.2 (left), 1 (middle), and 5 (right). The full and reduced models show consistent dynamics at each RT~. (TIF) [file pcbi.1013217.s003.tif]

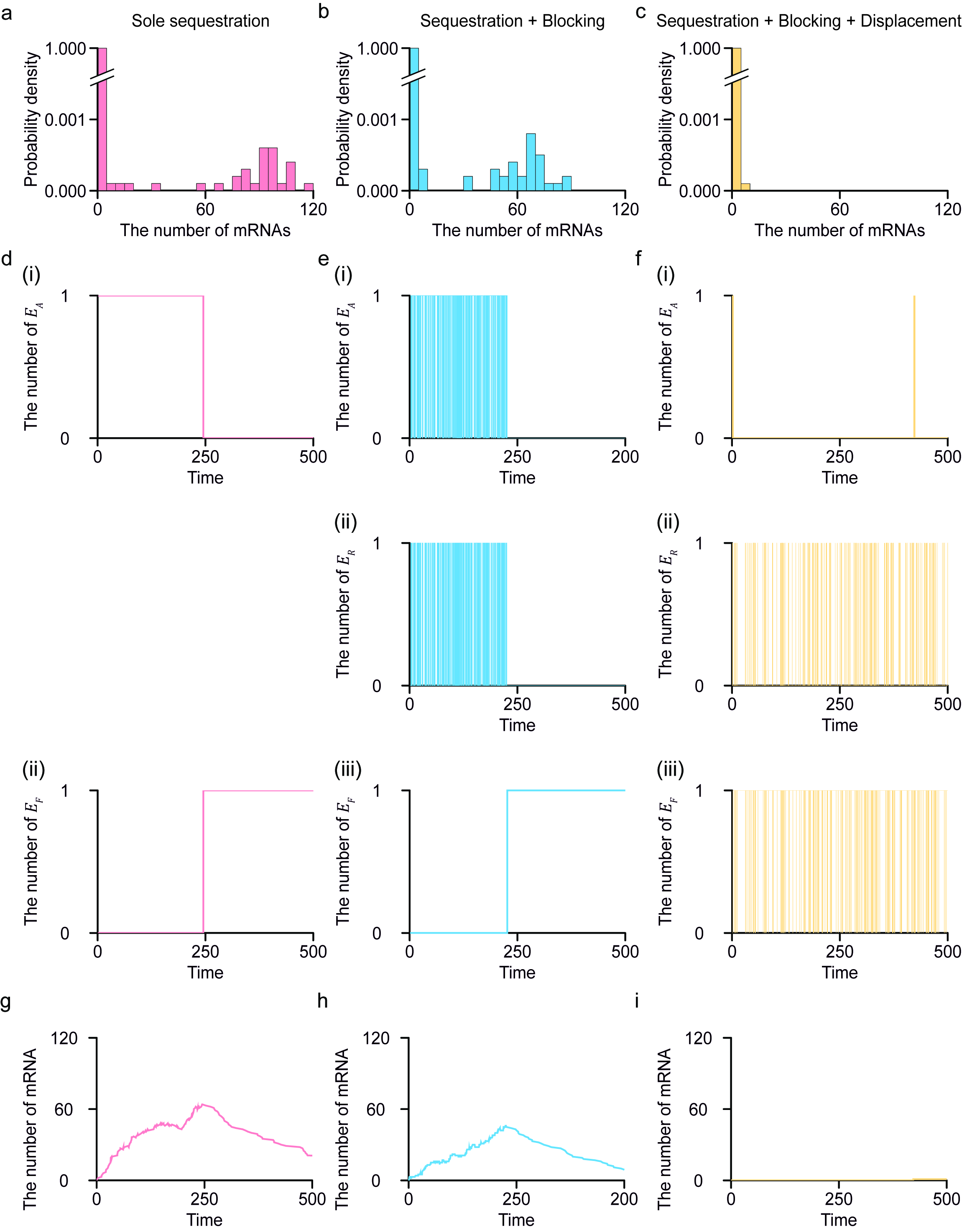

Supplement: S3 Fig — (a–c) Stationary distributions of mRNA copy numbers for the sole sequestration (a), combined sequestration and blocking (b), and combined sequestration, blocking, and displacement models (c), simulated using the parameters in Fig 3 at RT~=5. Despite the large number of repressors compared to the activators, both the sole sequestration model and the combined sequestration and blocking model exhibited bimodal mRNA distributions, with peaks at both low and high mRNA numbers, indicating that the activated DNA state persisted even in the presence of many repressors. In contrast, incorporation of displacement rendered the mRNA distribution unimodal, reflecting more consistent transcriptional repression. (d-i) Even under a large excess of repressors over activators, the sequestered activator can stochastically dissociate and rebind to DNA to form the active complex EA (d–f(i), at time 0). In the sole sequestration model (d), once the activator binds to DNA to form EA (d(i)), it remains bound for a long duration before dissociating into EF (d(ii)). This leads to continuous mRNA accumulation (g), and thus a bimodal mRNA distribution with a high Fano factor. In the combined sequestration and blocking model (e), EA rapidly transitions to the repressed state ER via blocking (e(i) and (ii)), and frequently interconverts between EA and ER until the activator dissociates from DNA to form EF (e(iii)). These frequent blocking events delay mRNA accumulation (h), and reduce the separation between the two peaks in the mRNA distribution, resulting in a lower Fano factor than in the sole sequestration model. In contrast, in the combined sequestration, blocking, and displacement model (f), EA rapidly transitions to ER (f(i) and (f(ii))) and displacement accelerates the transition from ER to EF (f(iii)), thereby preventing mRNA accumulation (i) and producing a unimodal mRNA distribution with a low Fano factor. (TIF) [file pcbi.1013217.s004.tif]

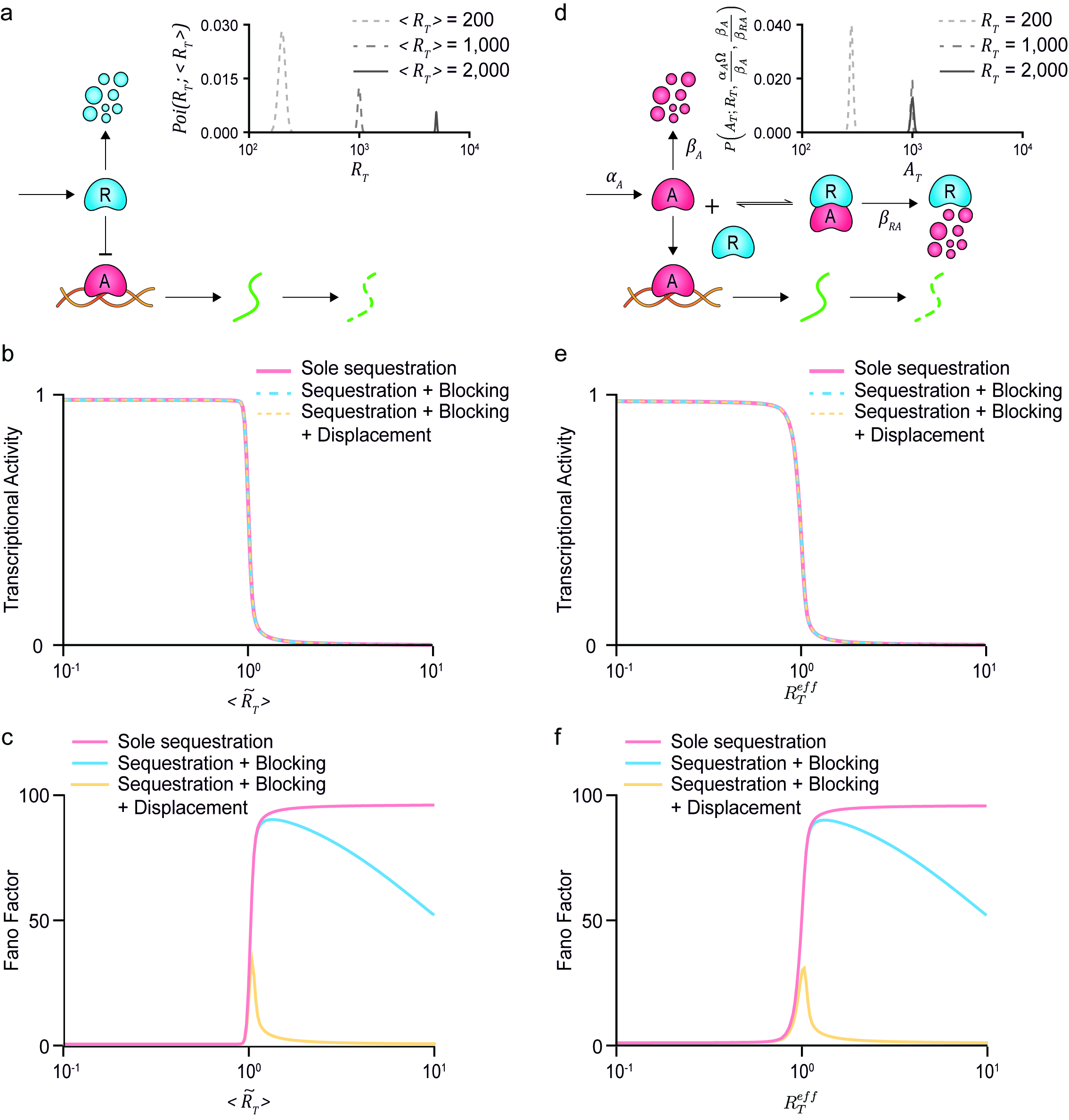

Supplement: S4 Fig — (a) When a repressor is regulated by a simple birth–death process, its copy number (RT) follows a Poisson distribution with the mean of . (b) In this case, the total mean of the mRNA copy number (M) can be calculated through the law of the total mean, E[M]=Eπ[E[M|RT]], where denotes the probability mass function of Poisson distribution with the mean of and E[M|RT] is the conditional mean of M for a given RT. As Eπ[E[M|RT]] can be calculated as the product of the production-to-degradation rate ratio (α/β) and the transcriptional activity TA(RT) in Table 2 (i.e., E[M|RT]=αβTA(RT)), the total mean of M becomes E[M]=Eπ[αβTA(RT)]=αβ∫TA(RT)π(RT;)dRT. Because the transcriptional activity TA(RT\)was set to be identical across all indirect repression models, their total mean mRNA numbers—and thus their effective transcriptional activities under repressor fluctuation (i.e., the total mean multiplied by β/α)—are also identical across models: sole sequestration (red), combined sequestration and blocking (blue), and combined sequestration, blocking, and displacement (yellow). (c) Similarly, the total variance of the mRNA copy number can be calculated through the law of the total variance, Var[M]=Varπ[E[M|RT]]+Eπ[Var[M|RT]], where Var[M|RT] is the conditional variance of M for a given RT. As Var[M|RT] can be calculated as the product of the product of E[M|RT] and the Fano factor FF(RT) in Table 2 (i.e., αβTA(RT)FF(RT)), the total variance of M becomes Var[M]=αβ∫(TA(RT)−E[M])2π(RT;)dRT+αβ∫TA(RT)FF(RT)π(RT;)dRT. Because the transcriptional activity TA(RT) was set to be identical across all indirect repression models, the first term is also the same among models. In contrast, the second term differs due to variations in the Fano factor FF(RT). Consequently, consistent with its lowest Fano factor FF(RT), the model combining sequestration, blocking, and displacement exhibits the smallest overall mRNA variance, and thus the lowest overall mRNA Fano factor, demonstr [file pcbi.1013217.s005.tif]

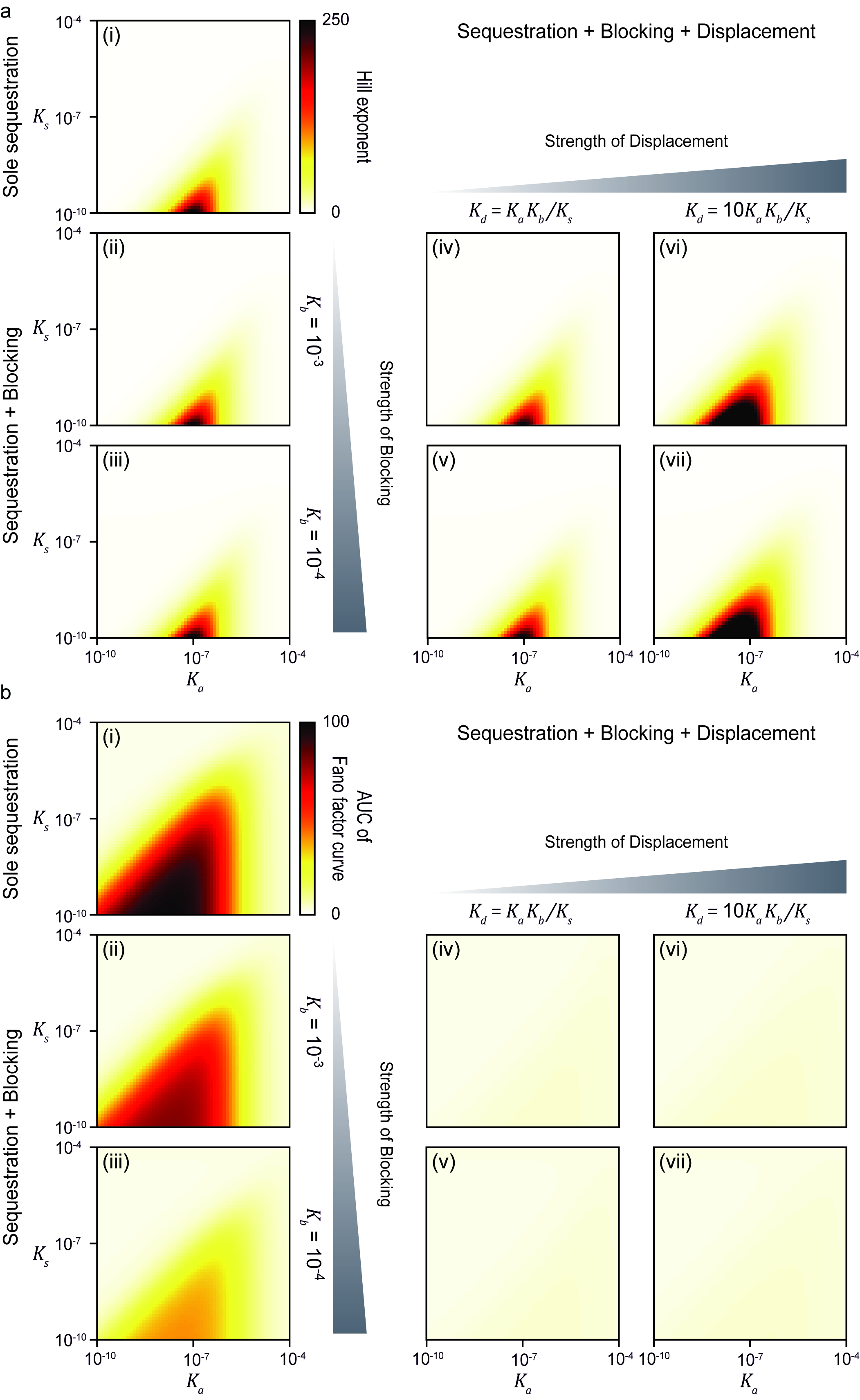

Supplement: S5 Fig — (a - b) Heatmaps showing the ultrasensitivity of transcriptional activity and the overall noise level, quantified by the Hill coefficient (a) and the area under the Fano factor curve (AUC) over a range of RT~ from 10−1 to 101 (b), respectively, by varying the dissociation constants Ka and Ks in the three indirect repression models. In the sole sequestration model, strong sequestration (i.e., Ks≪Ka) promoted high ultrasensitivity (a(i)) but was accompanied by elevated noise levels (b(i)). Incorporating blocking, with Kb=10−3, maintained a similar level of ultrasensitivity as in the sole sequestration case (a(ii)), while reducing the overall noise (b(ii)), indicating that the addition of blocking dampens fluctuations without compromising sensitivity. Further strengthening the blocking (Kb=10−4) lowered the noise even more (b(iii)) without loss of ultrasensitivity (a(iii)). Regardless of blocking strength, adding displacement, with its rate set to Kd=KaKb/Ks to maintain comparable ultrasensitivity (a(iv-v)), led to additional noise reduction (b(iv-v)), demonstrating cumulative noise suppression through cooperative multiple repressions. Moreover, stronger displacement (Kd=10KaKb/Ks) produced higher ultrasensitivity (a(vi-vii)) with sustained low noise (b(vi-vii)) compared to the weaker displacement (Kd=KaKb/Ks; a(iv-v) and b(iv-v)). Taken together, the sequential addition and strengthening of repression mechanisms progressively reduced noise while retaining or amplifying ultrasensitivity. (TIF) [file pcbi.1013217.s006.tif]
