# Supplementary material for: How cells tame noise while maintaining ultrasensitive transcriptional responses
Source: PLoS Comput Biol. 2025 Dec 11;21(12):e1013217. doi: 10.1371/journal.pcbi.1013217 (PMC12721554; doi:10.1371/journal.pcbi.1013217)
Supplement: Attachment — Submitted filename: Review Response_v2.pdf [file pcbi.1013217.s008.pdf]

We thank the reviewers for their constructive comments and suggestions for strengthening the presentation of our manuscript. In response, we have revised the manuscript. Below, we give our detailed responses to the reviewers' comments. The reviewers' comments appear in black, our responses in blue, and the manuscript in red (italic font indicates the revised text of the manuscript).

Reviewers' comments:

Reviewer's Responses to Questions

Reviewer #1: In the manuscript 'How Cells Tame Noise While Maintaining Ultrasensitive Transcriptional Responses', Jeong et al. performed mathematical modeling of transcriptional regulations with several types of mechanisms, including direct DNA binding, sequestration, blocking and displacement. They found that under biologically plausible parameter settings, the combination of three indirect repression mechanisms, but not other options, can maintain low noise of mRNA product while achieving high ultrasensitivity of the responses. They further showed that biological oscillators use this combination to achieve precise control of rhythmic dynamics in the precise of noise and large numbers of target genes. Overall, this is a rigorous study with very interesting observations. I have one suggestion for deepening the insight into the combination of the three mechanisms and a few others for improving the clarity of the manuscript.

1. The study has limited information on how the 3 indirect repression mechanisms synergize to achieve noise reduction and ultrasensitivity maintenance. While I understand this is a challenging question, readers would expect that the authors provide some insight since this is the main conclusion and the authors' models have provided a foundation for a mechanistic investigation.

1a) The authors can first look more carefully at the distributions of each species in Fig 2a with critical total repressor concentrations and deduce the activities of the 3 mechanisms.

1b) With 1a and other analysis, the authors can clarify whether the Blocking component is essential for

the performance by itself (e.g. there is a crucial amount of A-R-DNA complex) or merely serves as a mediator for displacement (e.g. a good performance is achieved when A is always displaced from DNA when B binds).

1c) By tuning the rate constants for the 3 mechanisms continuously and evaluating the performance of the model, the authors can investigate the effects of the 3 mechanisms at a deeper level.

[Response]

1a) We thank the reviewer for this thoughtful suggestion. We analyzed the distributions of mRNA numbers for each model (Supplementary Fig. 3a–c) using parameter sets corresponding to a molar ratio of repressors and activators equal to 5 ($\widetilde{R_T} = 5$). Under this condition, strong suppression would normally be expected. However, both the sole sequestration model and the combined sequestration and blocking model exhibited bimodal mRNA distributions with peaks at both low and high mRNA numbers (Supplementary Fig. 3a–b), indicating that an activated DNA state persisted even in the presence of many repressors. In contrast, when displacement was additionally incorporated (the combined sequestration, blocking, and displacement model), the mRNA distribution became unimodal (Supplementary Fig. 3c), reflecting consistent suppression of transcription. Consequently, this model showed the lowest variability, consistent with the reduced Fano factor observed in Figs. 2c and 3b.

1b) Even at a large molar ratio (i.e., when repressors outnumber activators), the sequestered activator can stochastically unbind from the repressor and rebind to DNA to form E_A (Supplementary Fig. 3d–f(i), at time 0). In the sole sequestration model, once E_A is formed, it persists for a long time (Supplementary Fig. 3d(i)) until the DNA-bound activator eventually unbinds from DNA itself and returns to the E_F state (Supplementary Fig. 3(ii)). This long-lasting E_A state causes a continuous increase in the mRNA copy number (Supplementary Fig. 3g), resulting in a bimodal mRNA distribution (Supplementary Fig. 3a) and thus a high Fano factor. In contrast, in the combined sequestration and blocking model, once E_A is formed (Supplementary Fig. 3e(i)), it rapidly transitions to the repressed state E_R via blocking (Supplementary Fig. 3e(ii)). Conversely, E_R can also revert to E_A when the repressor dissociates from the repressor–activator–DNA complex. These frequent transitions between

the E_A and E_R states continue until the activator eventually unbinds from DNA to form the E_F state (Supplementary Fig. 3e(iii)), similar to the sole sequestration model. Such frequent blocking events delay the accumulation of mRNA (Supplementary Fig. 3h), resulting in two peaks that are closer together in the mRNA distribution (Supplementary Fig. 3b) compared to the sole sequestration model (Supplementary Fig. 3a). In the combined sequestration, blocking, and displacement model, once E_A is formed (Supplementary Fig. 3f(i)), it quickly transitions to the repressed state E_R (Supplementary Fig. 3f(ii)) and then to the free E_F state through displacement (Supplementary Fig. 3f(iii)). This rapid displacement further suppresses the persistence of the E_A state, resulting in a unimodal mRNA distribution (Supplementary Fig. 3c) and thus a low Fano factor. In light of these observations, we have revised the Results section and included the new analyses in Supplementary Fig. 3, as follows:

Page 8 Line 169: *To further investigate noise levels under conditions of higher ultrasensitivity, we adjusted the dissociation constants of the indirect repression mechanisms to produce transcriptional activity with a Hill coefficient of 50 (Fig. 3a).* This elevated ultrasensitivity is accompanied by increased transcriptional noise (Fig. 2c and 3b). *This increase amplifies the differences among the models, which originate from stochastic transitions of DNA states* (Supplementary Fig. 3*) [22].*

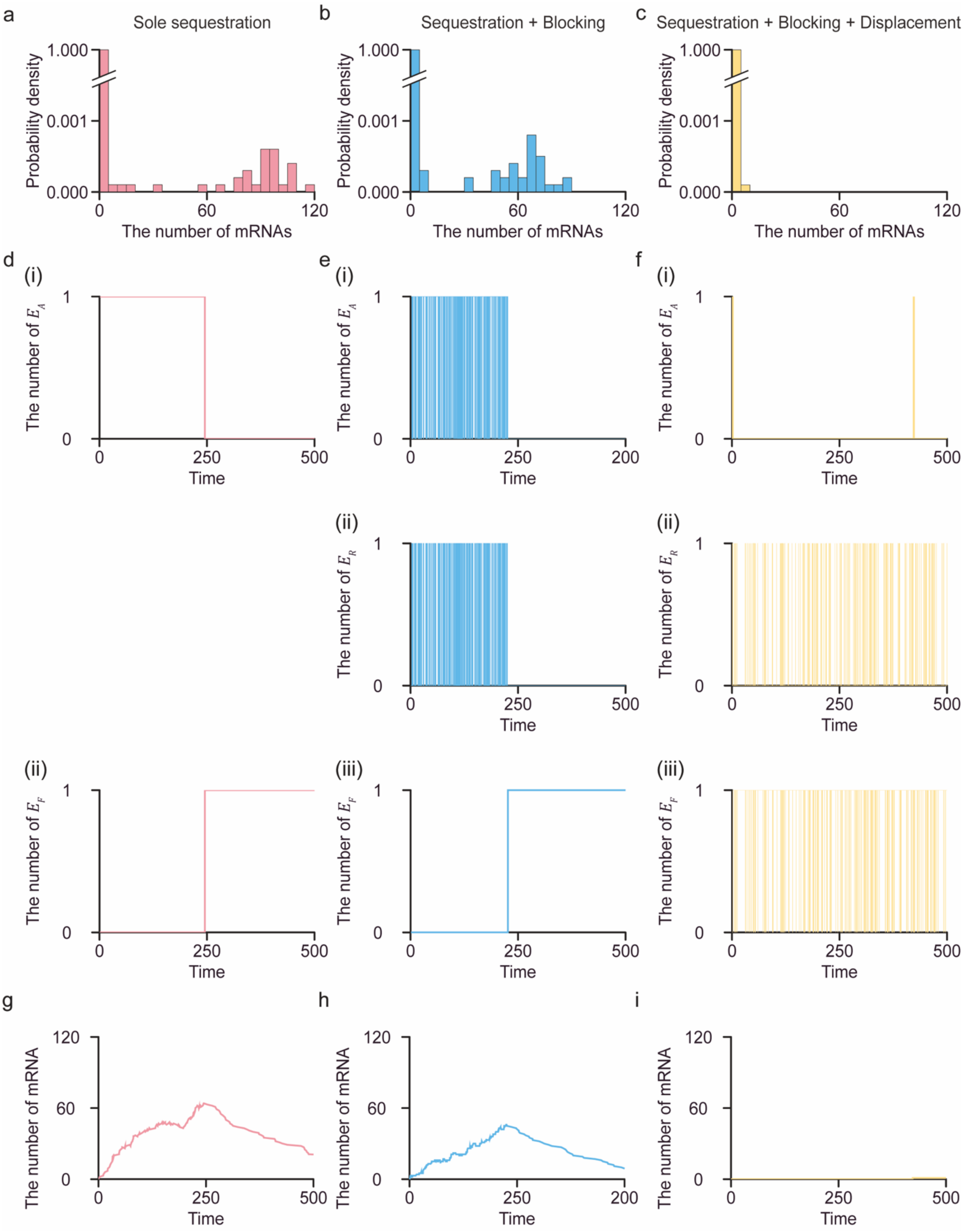

Supplementary Figure 3. Simulated time-series of DNA states and mRNA copy numbers, and stationary distribution of mRNA copy numbers across the three repression models.

(a–c) Stationary distributions of mRNA copy numbers for the sole sequestration (a), combined sequestration and blocking (b), and combined sequestration, blocking, and displacement models (c), simulated using the parameters in Fig. 3 at $\widetilde{R_T} = 5$. *Despite the large number of repressors compared to the activators, both the sole sequestration model and the combined sequestration and blocking model exhibited bimodal mRNA distributions, with peaks at both low and high mRNA numbers, indicating that the activated DNA state persisted even in the presence of many repressors. In contrast, incorporation of displacement rendered the mRNA distribution unimodal, reflecting more consistent transcriptional repression. (d-i) Even under a large excess of repressors over activators, the sequestered activator can stochastically dissociate and rebind to DNA to form the active complex* E_A *(d–f(i), at time 0). In the sole sequestration model (d), once the activator binds to DNA to form* E_A *(d(i)), it remains bound for a long duration before dissociating into* E_F *(d(ii)). This leads to continuous mRNA accumulation (g), and thus a bimodal mRNA distribution with a high Fano factor. In the combined sequestration and blocking model (e),* E_A *rapidly transitions to the repressed state* E_R *via blocking (e(i) and (ii)), and frequently interconverts between* E_A *and* E_R *until the activator dissociates from DNA to form* E_F *(e(iii)). These frequent blocking events delay mRNA accumulation (h), and reduce the separation between the two peaks in the mRNA distribution, resulting in a lower Fano factor than in the sole sequestration model. In contrast, in the combined sequestration, blocking, and displacement model (f),* E_A *rapidly transitions to* E_R *(f(i) and f(ii)) and displacement accelerates the transition from* E_R *to* E_F *(f(iii)), thereby preventing mRNA accumulation (i) and producing a unimodal mRNA distribution with a low Fano factor.*

Furthermore, in the new Supplementary Fig 3, the frequent transitions between the E_F and E_R states are observed in the combined sequestration, blocking, and displacement model (Supplementary Fig. 3f(ii) and f(iii)), implying that the E_R component primarily acts as an essential blocking element rather than as a mediator for displacement.

1c) We further investigate the effects of the three indirect repression mechanisms in a more continuous and systematic manner. To this end, we tuned the dissociation constants K_a and K_s, and evaluated the

sensitivity of transcriptional activity and the overall noise level, quantified by the Hill coefficient (Supplementary Fig. 5a) and the area under the Fano factor curve (AUC; Supplementary Fig. 5b), respectively. In the sole sequestration model, strong sequestration (i.e., $K_s \ll K_a$) was required to generate ultrasensitivity (Supplementary Fig. 5a(i)); however, this parameter regime also exhibited a high overall noise level (Supplementary Fig. 5b(i)), indicating that ultrasensitivity is inevitably accompanied by increased noise. When blocking was added with $K_b = 10^{-3}$, ultrasensitivity was maintained (Supplementary Fig. 5a(ii)), while the overall noise level was mitigated (Supplementary Fig. 5b(ii)), indicating that the blocking is capable of dampening fluctuations without loss of ultrasensitivity. Strengthening the blocking further ($K_b = 10^{-4}$) reduced noise even more, while maintaining comparable ultrasensitivity. Regardless of blocking strength, the inclusion of displacement preserved the overall transcriptional activity with $K_d = K_a K_b / K_s$ (Supplementary Fig. 5a(iv–v)), while further reducing the noise level (Supplementary Fig. 5b(iv–v)), indicating cooperative noise attenuation among the repression mechanisms. When the displacement strength was increased (i.e., $K_d = 100 K_a K_b / K_s$), transcriptional sensitivity was further enhanced (Supplementary Fig. 5a(vi-vii)), accompanied by a lower overall noise level (Supplementary Fig. 5b(vi-vii)) compared to the weaker displacement case (Supplementary Fig. 5a(iv-v) and 5b(iv-v)). Collectively, the addition of each repression mechanism reduced noise, and as the strength of repression increased, this reduction became more pronounced while ultrasensitivity was either maintained or further amplified. We have now added this result as a new Supplementary Fig. 5 and incorporated it into the revised Results section as follows:

Page 8 Line 169: *To further investigate noise levels under conditions of higher ultrasensitivity, we adjusted the dissociation constants of the indirect repression mechanisms to produce transcriptional activity with a Hill coefficient of 50 (Fig. 3a).* This elevated ultrasensitivity is accompanied by increased transcriptional noise (Figs. 2c and 3b). *This increase amplifies the differences among the models, which originate from stochastic transitions of DNA states (Supplementary Fig. 3) [22]. Furthermore, such attenuation of transcriptional noise—while maintaining comparable levels of ultrasensitivity through the addition of repression mechanisms—persists even when the numbers of activators and repressors fluctuate due to processes such as repressor birth–death dynamics and repressor-induced activator*

degradation (Supplementary Fig. 4). This effect is robust across a broad parameter range, and stronger repression further enhances noise suppression (Supplementary Fig. 5). Accordingly, under biologically relevant conditions, only the combination of sequestration, blocking, and displacement exhibits a low Fano factor ranging from 1 to 20—consistent with the experimentally observed range in mammalian cells—and is uniquely capable of reducing it to values close to 1 [37] (Fig. 3c).

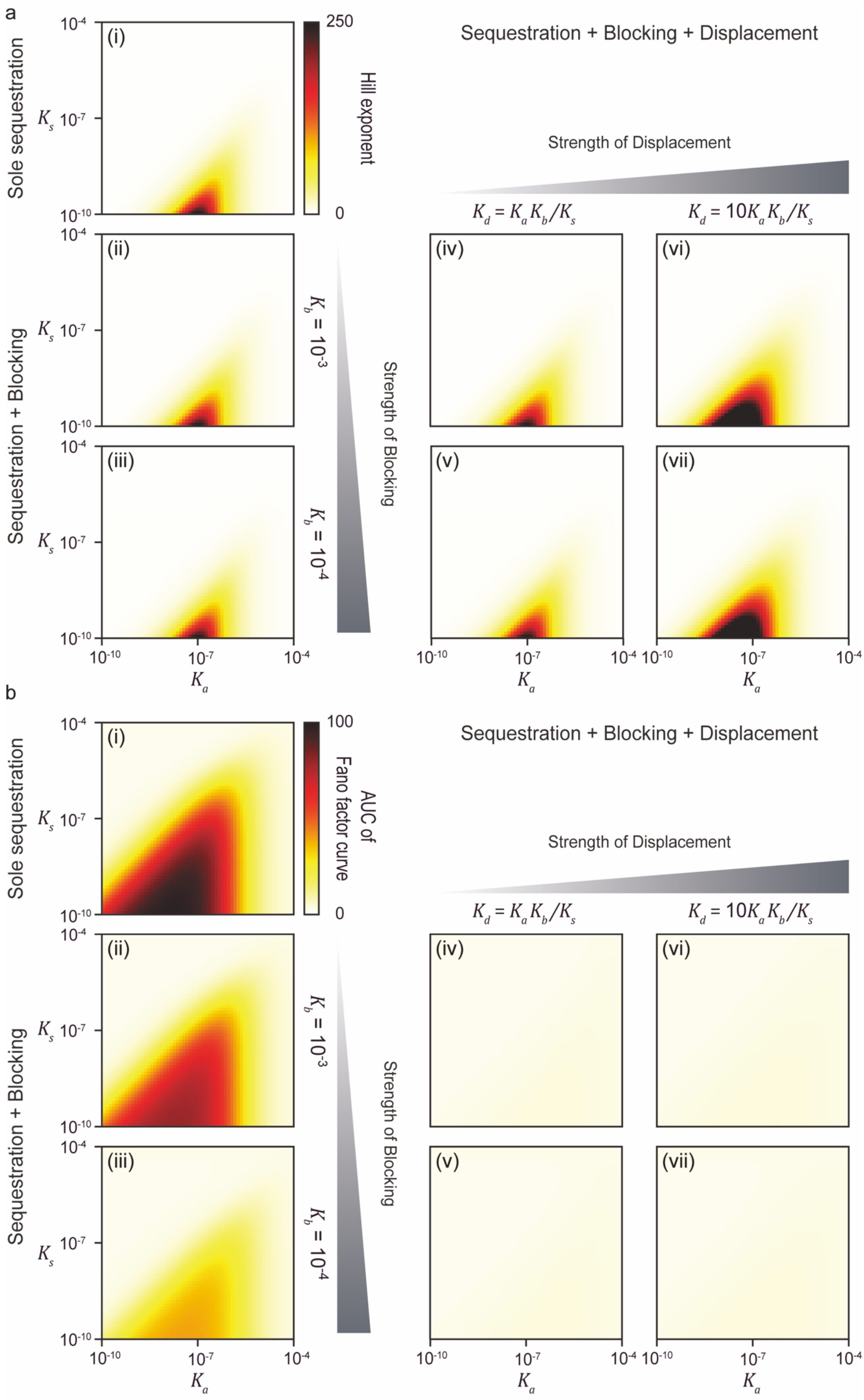
a
Sole sequestration
Sequestration + Blocking
Sequestration + Blocking + Displacement
Hill exponent
250
0
Strength of Displacement
Strength of Blocking
$K_d = K_a K_b / K_s$
$K_d = 10 K_a K_b / K_s$
$K_b = 10^{-3}$
$K_b = 10^{-4}$
(i) (ii) (iii) (iv) (v) (vi) (vii)
K_s
K_a
10^{-4} 10^{-7} 10^{-10}
b
Sole sequestration
Sequestration + Blocking
Sequestration + Blocking + Displacement
AUC of Fano factor curve
100
0
Strength of Displacement
Strength of Blocking
$K_d = K_a K_b / K_s$
$K_d = 10 K_a K_b / K_s$
$K_b = 10^{-3}$
$K_b = 10^{-4}$
(i) (ii) (iii) (iv) (v) (vi) (vii)
K_s
K_a
10^{-4} 10^{-7} 10^{-10}

Supplementary Figure 5. Systematic evaluation of the effects of indirect repression mechanisms on ultrasensitivity of transcriptional activity and overall noise level.

(a - b) Heatmaps showing the ultrasensitivity of transcriptional activity and the overall noise level, quantified by the Hill coefficient (a) and the area under the Fano factor curve (AUC) over a range of $\widetilde{R_T}$ *from* 10^{-1} *to* 10^{1} *(b), respectively, by varying the dissociation constants* K_a *and* K_s *in the three indirect repression models. In the sole sequestration model, strong sequestration (i.e.,* $K_s \ll K_a$*) promoted high ultrasensitivity (a(i)) but was accompanied by elevated noise levels (b(i)). Incorporating blocking, with* $K_b = 10^{-3}$*, maintained a similar level of ultrasensitivity as in the sole sequestration case (a(ii)), while reducing the overall noise (b(ii)), indicating that the addition of blocking dampens fluctuations without compromising sensitivity. Further strengthening the blocking (*$K_b = 10^{-4}$*) lowered the noise even more (b(iii)) without loss of ultrasensitivity (a(iii)). Regardless of blocking strength, adding displacement, with its rate set to* $K_d = K_a K_b / K_s$ *to maintain comparable ultrasensitivity (a(iv-v)), led to additional noise reduction (b(iv-v)), demonstrating cumulative noise suppression through cooperative multiple repressions. Moreover, stronger displacement (*$K_d = 10 K_a K_b / K_s$*) produced higher ultrasensitivity (a(vi-vii)) with sustained low noise (b(vi-vii)) compared to the weaker displacement (*$K_d = K_a K_b / K_s$*; a(iv-v) and b(iv-v)). Taken together, the sequential addition and strengthening of repression mechanisms progressively reduced noise while retaining or amplifying ultrasensitivity.*

2. The scope of the work and the proper introduction of the system with biological context need to be better presented at the beginning. For example, 'One of the most well-known mechanisms that produce ultrasensitive responses is cooperative binding, in which transcriptional repressors bind cooperatively to multiple sites on DNA.' appears as the second sentence of the manuscript, but cooperativity and ultrasensitive responses do not even have to involve transcription in biology: many forms of binding involving small molecules, proteins and RNAs can produce those phenomena.

[Response] We thank the reviewer for this constructive comment. We agree that ultrasensitivity and cooperativity are general principles in biology and are not limited to transcriptional regulation. They can also arise in diverse contexts such as protein–protein interactions, protein–RNA interactions, and small-molecule binding. Our intention in the original manuscript was to highlight transcriptional regulation as a particularly well-studied and biologically relevant setting in which these mechanisms play a central role. To clarify this point and to better frame the system within its broader biological context, we have revised the Introduction section with additional references as follows:

Page 3 Line 54: Ultrasensitivity, characterized by a sharp output change in response to a small variation in input, underlies many essential regulatory functions in biological systems, including signal amplification, bistability, and oscillatory dynamics [1-4]. One of the most well-known mechanisms that produce ultrasensitive responses is cooperative binding, *which is broadly observed across biology, in phenomena such as receptor-ligand binding [5, 6], protein–RNA interactions [7], and small-molecule binding [8]. A prominent example in transcriptional regulation is the cooperative binding of repressors to multiple DNA sites, which* enables the formation of sharp transcriptional switches, where small changes in repressor concentration lead to abrupt transcriptional repression *[9, 10]*. While cooperative binding has *traditionally been considered a primary strategy for achieving ultrasensitivity*, recent theoretical studies have highlighted alternative approaches *[11-20]*.

3. Line 108: 'To investigate how much faster _f and _r attenuate transcriptional noise…'. This part of the sentence is confusing. Do these parameters accelerate noise reduction, or are the authors investigating the range of them that would reduce noise?

[Response] We apologize for the confusion. To clarify, an increase in both k_f and k_r (corresponding to k_a, k_b, k_s, and k_d in the case of indirect mechanisms) consistently reduces noise levels. This behavior is evident in heatmaps (Fig. 1e, 2d, and 3c), where the color approaches the white region as the value of k_f increases, indicating minimal noise (i.e., a Fano factor of 1). This is consistent with the

equation for the Fano factor, which takes the form of $1 + (1/k_f)$ multiplied by a rational function of $K_a = k_a/k_f$, $K_b = k_b/k_f$, $K_s = k_s/k_f$, and $K_d = k_d/k_f$ (Table 2). Accordingly, the Fano factor converges to 1 as both binding and unbinding rates increase simultaneously. Now, we have revised the manuscript to clarify this as follows:

Page 5 Line 115: To investigate how *increasing the values of* k_f and k_r attenuate*s* transcriptional noise, we simulated the stationary distribution of mRNA copy numbers (Fig. 1d) *using the Gillespie algorithm (see Methods) [27]*.

4. Each modeling framework (ODE? CME? Stochastic simulations?) needs to be concisely introduced before the first result plot (e.g. Fig 1b) in addition to the wiring diagram.

[Response] We thank the reviewer for the suggestion. To derive the equations for transcriptional activity and the Fano factor, we used the CME framework. As suggested, this is now briefly stated before Fig. 1b, with a reference to the Methods section for detailed derivations as follows:

Page 5 Line 104: When all sites are occupied, transcription is inhibited, but it remains active when at least one site is unoccupied, producing mRNA at a rate of α, which degrades at a rate of β (Table 1). Thus, the transcriptional activity (i.e., the probability at which the transcription is active)*, derived using the chemical master equation (CME) framework (see Methods),* decreases as the number of repressors (R_T) increases.

Furthermore, the simulations conducted in response to the first comment, as well as those for the stationary distribution of mRNA copy numbers in Figure 1, were performed using the Gillespie algorithm. We now clarify this point in the revised manuscript as follows:

Page 5 Line 111: This ultrasensitivity is maintained as long as the dissociation constant $K_r = k_r/k_f$ remains fixed, even when k_f varies. However, the noise level changes when k_f varies. For instance, as $k_f = 6 \times 10^2$ is increased to $k_f = 6 \times 10^5$, while keeping $K_r = 10^{-2}$, the level of ultrasens*i*tivity

does not change (Fig. 1b), but the noise level quantified with the Fano factor (i.e., variance/mean) of *the* mRNA copy numbers considerably decreases (Fig. 1c). To investigate how *increasing the values of* k_f and k_r attenuate*s* transcriptional noise, we simulated the stationary distribution of mRNA copy numbers (Fig. 1d) *using the Gillespie algorithm (see Methods) [27]*.

5. Many plots in this work have overlapped curves. The visualization of such data needs to be improved otherwise it is difficult for readers to distinguish different models. I suggest that the authors use different line widths for different models and use darker and thinner curves for those in front.

[Response] We sincerely appreciate the reviewer's comment. Indeed, several curves in the transcriptional activity plots (Fig. 1b, 2b, and 3a) overlapped, making it difficult to distinguish between models. Following the reviewer's suggestion, we have improved the visualization by adjusting line widths and using thinner lines with wider dot spacing for the front layers, which makes the different models easier to distinguish. In addition, we have increased the figure sizes to further enhance clarity, as follows:

Figure 1

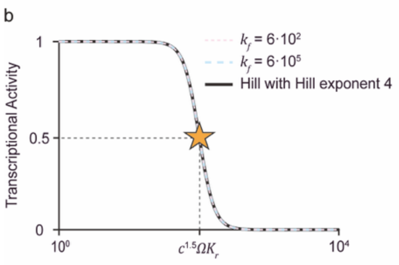

Figure 2

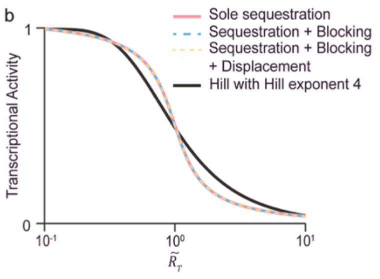

Figure 3

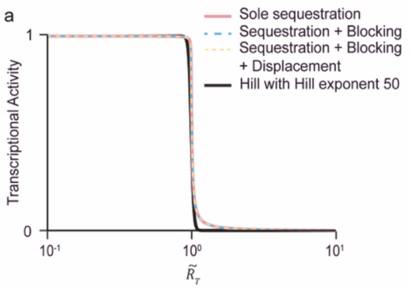

6. Please clarify in the text what type of cell the cell volume parameter corresponds to. Similarly, the ranges of mRNA half lives for yeast cells and mammalian cells differ significantly. The cell types need to be specified when the authors state the range of the parameter.

[Response] We appreciate the reviewer's valuable feedback. Most parameters used in this study were obtained from typical mammalian cells, while several rate constants were derived from *in vitro* or *in silico* theoretical studies. Specifically, cell volume (Ω) was referenced from typical mammalian cells, with its value in the range of 10^{-12} to 10^{-11} l [R1]. The mRNA half-life was taken from mouse embryonic stem cells and human whole blood, where their measurements report values ranging from less than 1 hour to up to 16.4 hours [R2, R3]. The concentration of activators (A_T/Ω) in *H. sapiens* cell lines typically lies between $10^{-9}M$ and $10^{-7}M$ [R1]. For the binding rate (k_f), it was theoretically estimated by relating the dissociation constant to the interdependence between binding and unbinding rates, while considering physical and diffusion constraints. These frameworks predict the protein-protein binding rate of $10^6 \sim 10^7 M^{-1}s^{-1}$ [R4]. Indeed, the binding rate between p53 and Mdm2 ($9.2 \pm 0.04 \times 10^6 M^{-1}s^{-1}$) determined from *in silico* molecular dynamics simulations [R5], and that of the transcriptional activator NF-κB binding to ($10^6 M^{-1}s^{-1}$) measured *in vitro* in *E. coli* BL21 (DE3) expressing human NF-κB [R6], both fall within this range. Based on these clarifications, we have added a new table that explicitly specifies the corresponding cell types for each parameter, as follows:

Page 6 Line 125:

However, achieving this requires biologically unrealistic fast binding and unbinding rates. Specifically, with a biologically relevant binding rate $k_f \Omega^{-1} = 6 \times 10^{-6} \sim 6 \times 10^{-5}$ $ymol^{-1}min^{-1}$ *[24, 29, 30]*, where $\Omega = 10^7 aL$ is the volume of the cell [23], and the mRNA half-life is 0.5 ~ 16.4 hours (i.e., 30 ~ 984 min; *Table 2*) [31, 32], the Fano factor is greater than 40 (Fig. 1e, gray box). Taken together, while increasing binding rates can minimize noise, the cooperative binding mechanism remains highly noisy under physiological conditions.

Page 7 Line 161:

However, within the biologically relevant range of binding rate and mRNA half-life, the Fano factor still remains high, with a minimum of about 4 (*Table 2 and* Fig. 2d, top row). In this case, we used the number of activators $A_T = 10^3$.

Page 41 Line 755:

Table 2. The references of parameter values used for model simulations.

Cell type	***Parameter (definition)***	***Reported range***	***References***
In vitro (Cell host: human NF-κB and IκBα expressed in E. coli BL21 DE3); N/A – Theoretical / literature-based study; In silico molecular dynamics simulation	k_f *(binding rate)*	$10^6 \sim 10^7\ M^{-1}s^{-1}$	*Williamson, 2023; Bergqvist et al., 2009; Zou et al., 2020*
Typical mammalian cells	*Ω (cell volume)*	$10^{-12} \sim 10^{-11} l$	*Milo et al., 2016*
Mouse embryonic stem cells, human whole blood	*ln 2 / β (mRNA half-life)*	*0.5 (less than 1 h) ~ 16.4 h*	*Sharova et al., 2009; Wang & Liu, 2022*
H. sapiens cell lines	A_T/Ω *(transcription factor concentration)*	$10^{-9} \sim 10^{-7} M$	*Milo et al., 2016*

7. For the NF-kappaB model, it is known that the outputs from the system are very noisy (e.g. https://doi.org/10.1038/s41467-025-60901-3), and only a small subset of the outputs have clear rhythmic patterns. How can the authors justify the importance of noise reduction and ultrasensitivity in this example?

[Response] We thank the reviewer for raising this important point. Unlike circadian clocks that sustain rhythmicity under constant conditions, oscillations in systems such as NF-κB and p53 typically occur only in response to sufficiently strong stimuli or cellular damage [R7, R8]. In these systems, the onset and regularity of oscillations depend on the magnitude of upstream signaling, and therefore, cell-to-cell variability in the amplitude or duration of input signals can lead to heterogeneous responses across the population. The reported heterogeneity in NF-κB [R9] and p53 dynamics [R8] may thus arise primarily

from variability in damage or stimulus strength, rather than from intrinsic noise in the core regulatory circuit. To clarify this point, we have modified the Discussion section as follows:

Page 11 Line 261:

In summary, our findings suggest that combining indirect repression mechanisms offers a resource-efficient strategy for achieving robust, rhythmic gene expression in noisy cellular environments—potentially explaining why sequestration, blocking, and displacement often co-occur in natural biological oscillators.

This strategy is exemplified in systems such as NF-κB oscillators, which often exhibit heterogeneous single-cell oscillatory responses [55]. This heterogeneity primarily results from differences in upstream stimulus strength or duration, such as variations in inflammatory stimuli TNF-a pulse strength or duration that modulate the timing of NF-κB activation [56]. Similarly, in the p53-MDM2 oscillators, cell-to-cell variability in oscillatory dynamics is induced by differences in DNA damage or oncogenic stress [57]. These demonstrate that variability in input stimuli primarily determines when and how cells cross the activation threshold, required to initiate oscillations [58, 59]. Therefore, our findings are primarily relevant to the regime where oscillations are triggered by sufficiently strong inputs, and to how the identified repression mechanisms help maintain coherent and stable rhythms of initiated oscillations. Extending this framework to variable input conditions, where upstream stimuli couple with repression strategies thus presents a compelling future research direction for understanding population-level transcriptional dynamics.

Given such heterogeneity, our findings focused on the regime where the input signal is sufficiently strong to induce oscillations. Under such conditions, the mechanisms we identified—those that enhance noise robustness and ultrasensitivity—can help ensure that once oscillations are triggered, they remain coherent and stable across time. This implies that the proposed mechanisms contribute to maintaining reliable oscillatory behavior in response to substantial damage, which is crucial for the proper

functioning of stress-responsive systems such as NF-κB and p53. Now, we have highlighted this point to the Result section as follows:

Page 8 Line 185:

The combination of sequestration, blocking, and displacement *constitutes a core regulatory logic shared* across diverse biological oscillators. In the mammalian circadian clock, the PER–CRY complex sequesters CLOCK–BMAL1, displaces it from DNA, *and CRY further blocks its transcriptional activity* [21, 38-44] (Fig. 4a). In the NF-κB oscillator, IκB sequesters NF-κB in the cytoplasm, *and* upon external stimulation, *its degradation allows NF-κB to activate transcription; this activation is subsequently* suppressed as IκB displaces NF-κB from DNA [29, 45] (Fig. 4b). In the p53–Mdm2 oscillator, Mdm2 sequesters p53 away from DNA, blocks its transcriptional activity, and displaces it through cooperative interactions with corepressors [46, 47] (Fig. 4c). *Although these systems differ in their biological roles—circadian rhythms must sustain precise oscillations under constant conditions, whereas NF-κB and p53 oscillations are transient and stimulus-induced—they employ the same regulatory strategy to achieve functional robustness. The coordinated action of sequestration, blocking, and displacement sharpens the input–output response, creating ultrasensitivity while buffering against fluctuations in upstream signals. We hypothesized that this noise-resistant ultrasensitive switch (Fig. 4d, left) enables strong oscillatory responses and precise timing of transitions between transcriptional on and off states (Fig. 4d, right) [1, 3, 4, 14], thereby ensuring coherent and stable oscillations once they are triggered.*

To validate this hypothesis, we constructed a transcriptional negative feedback loop (NFL) model (Fig. 4e and Table 1). In the NFL model, …

8. MDM2 (R) is known to promote p53 degradation (A), and this is considered a crucial part of the negative feedback. This mechanism does not seem to be included in the authors' model, so it is unclear whether it plays a role in noise reduction and whether its presence would affect the authors' conclusion.

[Response] We thank the reviewer for this insightful comment. Indeed, when the repressor (MDM2) binds to and promotes the degradation of the activator (p53), the activator degradation rate becomes nonlinear in its copy number. Specifically, in our model, activators are produced at rate α_A, degraded at rate $\beta_A A/\Omega$, and further degraded in the form of a repressor–activator complex at rate $\beta_{RA} R_A/\Omega$ (Supplementary Fig. 4(a)), where $A(A_T;R_T) = \frac{A_T - R_T - \Omega K_s + \sqrt{(A_T - R_T - \Omega K_s)^2 + 4\Omega K_s A_T}}{2} \approx \begin{cases} 0, \ A_T \le R_T \\ A_T - R_T, A_T > R_T \end{cases}$, and $R_A(A_T;R_T) = \frac{A_T + R_T + \Omega K_s - \sqrt{(A_T - R_T - \Omega K_s)^2 + 4\Omega K_s A_T}}{2} \approx \begin{cases} A_T, \ A_T \le R_T \\ R_T, A_T > R_T \end{cases}$ represent the number of free activators and repressor–activator complexes, respectively, as functions of the activator copy number (A_T) for a given R_T. Then, the corresponding chemical master equation of this system is given by

$$\frac{dP(A_T)}{dt} = \alpha_A P(A_T - 1) + \left(\beta_A \frac{A(A_T+1)}{\Omega} + \beta_{RA} \frac{R_A(A_T+1)}{\Omega}\right) P(A_T + 1) - \left(\alpha_A + \beta_A \frac{A(A_T)}{\Omega} + \beta_{RA} \frac{R_A(A_T)}{\Omega}\right) P(A_T),$$

where $P(A_T)$ is the probability that the total number of activators is A_T. At the stationary distribution, the equation becomes:

$$0 = \alpha_A P(A_T - 1) + \left(\beta_A \frac{A(A_T+1)}{\Omega} + \beta_{RA} \frac{R_A(A_T+1)}{\Omega}\right) P(A_T + 1) - \left(\alpha_A + \beta_A \frac{A(A_T)}{\Omega} + \beta_{RA} \frac{R_A(A_T)}{\Omega}\right) P(A_T) \ ,$$

and

$$(A_T + 1) = \frac{\frac{\alpha_A \Omega}{\beta_{RA}}}{\frac{\beta_A}{\beta_{RA}} A(A_T;R_T) + R_A(A_T;R_T)} P(A_T) \approx \begin{cases} \frac{\frac{\alpha_A \Omega}{\beta_{RA}}}{A_T} P(A_T) \\ \frac{\frac{\alpha_A \Omega}{\beta_{RA}}}{\frac{\beta_A}{\beta_{RA}} A_T + \left(1 - \frac{\beta_A}{\beta_{RA}}\right) R_T} P(A_T) \end{cases} .$$

As a result, we can compute the distribution of A_T recursively. Notably, when the degradation rates of activator solely and in the form of a repressor-activator complex are same (i.e., $\beta_A = \beta_{RA}$), A_T follows simple Poisson. In contrast, when repressor promote the degradation of activator by binding (i.e., $\beta_A < \beta_{RA}$), the stationary distribution of A_T deviates from a simple Poisson form.

Nevertheless, the total variability of mRNA does not depend on the specific distributional form of the activator, according to the laws of total mean and variance. Specifically, by the law of total mean, the overall mean of the mRNA number is given by $E_\pi\left[E[M|A_T]\right]$, where M is the mRNA number, A_T is the total number of repressors, and π denotes the probability density function of distribution that A_T follows. For a given A_T, the conditional mean of M (i.e., $E[M|A_T]$) can be calculated as the product of the production-to-degradation rate ratio (α/β) and the transcriptional activity $TA(A_T)$ from Table 2; that is, $E[M|A_T] = \frac{\alpha}{\beta}TA(A_T)$. Hence, the overall mean of the mRNA number is given by

$$E[M] = E_\pi\left[\frac{\alpha}{\beta}TA(A_T)\right] = \frac{\alpha}{\beta}\int TA(A_T)\pi(A_T)dA_T.$$

Because the transcriptional activity was set to be identical across all indirect repression models (Fig. 3a), their overall mean mRNA levels are also identical, regardless of π (Supplementary Fig 4e).

Next, according to the law of total variance, the overall variance of the mRNA number is given by $Var[M] = Var_\pi\left[E[M|A_T\ ]\right] + E_\pi\left[Var[M|A_T]\right]$. For given A_T, the conditional variance of M (i.e., $Var[M|A_T]$) can be expressed as the product of $E[M|A_T\ ]$ and the Fano factor $FF(A_T)$ from Table 2; that is, $Var[M|A_T] = \frac{\alpha}{\beta}TA(A_T)FF(A_T)$. Hence, the overall variance of the mRNA number is given by

$$Var[M] = Var_\pi\left[\frac{\alpha}{\beta}TA(A_T)\right] + E_\pi\left[\frac{\alpha}{\beta}TA(A_T)FF(A_T)\right] = \frac{\alpha}{\beta}\int (TA(A_T) - E[M])^2\pi(A_T)dA_T + \frac{\alpha}{\beta}\int TA(A_T)FF(A_T)\pi(A_T)dA_T.$$

Because of identical transcriptional activities across all indirect repression models (Fig. 3a), the first term $Var_\pi\left[\frac{\alpha}{\beta}TA(A_T)\right]$ is the same for all. In contrast, their Fano factors—and thus the second term $E_\pi\left[\frac{\alpha}{\beta}TA(A_T)FF(A_T)\right]$—differ among models (Fig. 3b). Consequently, consistent with its lowest Fano factor $FF(A_T)$, the model combining sequestration, blocking, and displacement exhibits the smallest overall mRNA variance, and thus the smallest overall mRNA Fano factor (Supplementary Fig. 4f). These results demonstrate that repressor-mediated destabilization of activators does not influence the

noise reduction achieved by the combined sequestration, blocking, and displacement mechanism. These findings are now presented in Supplementary Fig. 4, and the Results section has been revised accordingly.

Page 8 Line 169: *To further investigate noise levels under conditions of higher ultrasensitivity, we adjusted the dissociation constants of the indirect repression mechanisms to produce transcriptional activity with a Hill coefficient of 50 (Fig. 3a).* This elevated ultrasensitivity is accompanied by increased transcriptional noise (Figs. 2c and 3b). *This increase amplifies the differences among the models, which originate from stochastic transitions of DNA states (Supplementary Fig. 3) [22]. Furthermore, such attenuation of transcriptional noise—while maintaining comparable levels of ultrasensitivity through the addition of repression mechanisms—persists even when the numbers of activators and repressors fluctuate due to processes such as repressor birth–death dynamics and repressor-induced activator degradation (Supplementary Fig. 4). This effect is robust across a broad parameter range, and stronger repression further enhances noise suppression (Supplementary Fig. 5). Accordingly, under biologically relevant conditions, only the combination of sequestration, blocking, and displacement exhibits a low Fano factor ranging from 1 to 20—consistent with the experimentally observed range in mammalian cells—and is uniquely capable of reducing it to values close to 1 [37] (Fig. 3c).*

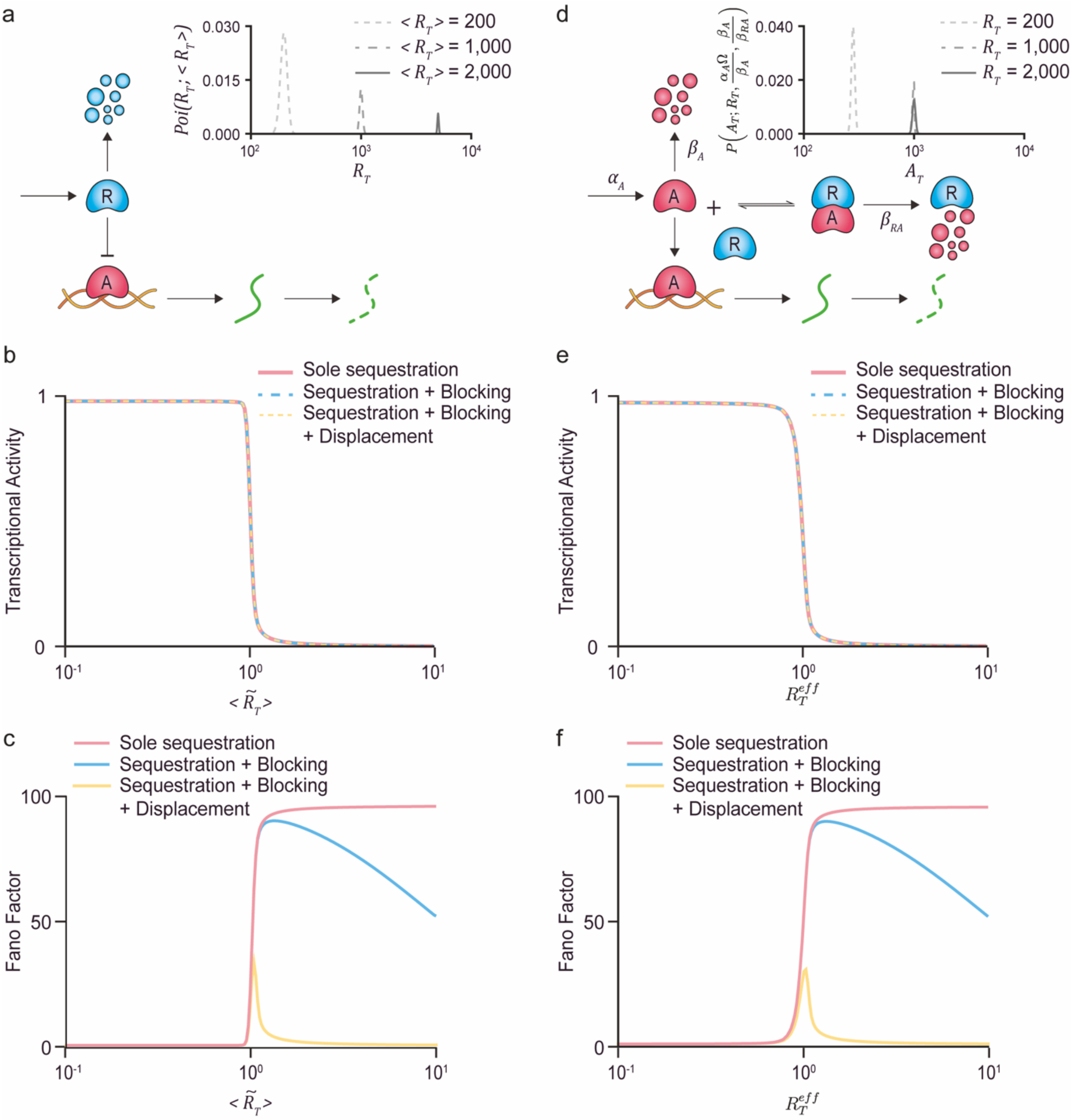

Supplementary Figure 4. Despite fluctuations in repressor or activator copy number, the combined sequestration, blocking, and displacement model maintains robustness of noise reduction.

(a) When a repressor is regulated by a simple birth–death process, its copy number (R_T) follows a Poisson distribution with the mean of $< R_T >$. (b) In this case, the total mean of the mRNA copy number (M) can be calculated through the law of the total mean, $E[M] = E_\pi[E[M|R_T]]$, where denotes the probability mass function of Poisson distribution with the mean of $< R_T >$ and $E[M|R_T]$ is the

conditional mean of M for a given R_T. As $E_\pi[E[M|R_T]]$ can be calculated as the product of the production-to-degradation rate ratio (α/β) and the transcriptional activity $TA(R_T)$ in Table 2 (i.e., $E[M|R_T] = \frac{\alpha}{\beta}TA(R_T)$), the total mean of M becomes $E[M] = E_\pi\left[\frac{\alpha}{\beta}TA(R_T)\right] = \frac{\alpha}{\beta}\int TA(R_T)\pi(R_T; < R_T >)dR_T$. Because the transcriptional activity $TA(R_T)$ was set to be identical across all indirect repression models, their total mean mRNA numbers—and thus their effective transcriptional activities under repressor fluctuation (i.e., the total mean multiplied by β/α)—are also identical across models: sole sequestration (red), combined sequestration and blocking (blue), and combined sequestration, blocking, and displacement (yellow). (c) Similarly, the total variance of the mRNA copy number can be calculated through the law of the total variance, $Var[M] = Var_\pi[E[M|R_T]] + E_\pi[Var[M|R_T]]$, where $Var[M|R_T]$ is the conditional variance of M for a given R_T. As $Var[M|R_T]$ can be calculated as the product of the product of $E[M|R_T]$ and the Fano factor $FF(R_T)$ in Table 2 (i.e., $\frac{\alpha}{\beta}TA(R_T)FF(R_T)$), the total variance of M becomes $Var[M] = \frac{\alpha}{\beta}\int (TA(R_T) - E[M])^2\pi(R_T; < R_T >)dR_T + \frac{\alpha}{\beta}\int TA(R_T)FF(R_T)\pi(R_T; < R_T >)dR_T$. Because the transcriptional activity $TA(R_T)$ was set to be identical across all indirect repression models, the first term is also the same among models. In contrast, the second term differs due to variations in the Fano factor $FF(R_T)$. Consequently, consistent with its lowest Fano factor $FF(R_T)$, the model combining sequestration, blocking, and displacement exhibits the smallest overall mRNA variance, and thus the lowest overall mRNA Fano factor, demonstrating its robustness even under fluctuations in repressor copy number. (d) The number of activators can also fluctuate, when repressors bind to and promote the degradation of activators, as observed in systems such as the p53–MDM2 oscillator. To incorporate such fluctuations, we modeled the birth-death dynamics of the activator. Specifically, activators are produced at a rate α_A, degraded as free activators at a rate $\beta_A A/\Omega$, and further degraded in the form of repressor–activator complexes at a rate $\beta_{RA}R_A/\Omega$. Here, $A(A_T; R_T) = \frac{A_T - R_T - \Omega K_S + \sqrt{(A_T - R_T - \Omega K_S)^2 + 4\Omega K_S A_T}}{2} \approx \begin{cases} 0, & A_T \le R_T \\ A_T - R_T, & A_T > R_T \end{cases}$, and $R_A(A_T; R_T) = \frac{A_T + R_T + \Omega K_S - \sqrt{(A_T - R_T - \Omega K_S)^2 + 4\Omega K_S A_T}}{2} \approx \begin{cases} A_T, & A_T \le R_T \\ R_T, & A_T > R_T \end{cases}$ represent the number of free

activators and repressor–activator complexes, respectively, as functions of the activator copy number (A_T) for a given R_T. Then, the probability distribution of A_T is governed by the following CME:

$$\frac{dP(A_T)}{dt} = \alpha_A P(A_T - 1) + \left(\beta_A \frac{A(A_T+1)}{\Omega} + \beta_{RA} \frac{R_A(A_T+1)}{\Omega}\right) P(A_T + 1) - \left(\alpha_A + \beta_A \frac{A(A_T)}{\Omega} + \beta_{RA} \frac{R_A(A_T)}{\Omega}\right) P(A_T),$$

where $P(A_T)$ denotes the probability that the total number of activators is A_T, At stationary distribution (i.e., $\frac{dP(A_T)}{dt} = 0$), the CME satisfies $P(A_T + 1) = \frac{\frac{\alpha_A \Omega}{\beta_{RA}}}{\frac{\beta_A}{\beta_{RA}} A(A_T; R_T) + R_A(A_T; R_T)} P(A_T) \approx \begin{cases} \frac{\frac{\alpha_A \Omega}{\beta_{RA}}}{A_T} P(A_T) \\ \frac{\frac{\alpha_A \Omega}{\beta_{RA}}}{\frac{\beta_A}{\beta_{RA}} A_T + \left(1 - \frac{\beta_A}{\beta_{RA}}\right) R_T} P(A_T) \end{cases}$. Thus, the stationary distribution of A_T can be calculated recursively, and is determined by $\alpha_A \Omega / \beta_{RA}$ and β_{RA}/β_A, which represent the effective number of the activator and the relative degradation activator mediated by the repressor, respectively. Here, we simulated with $\alpha_A \Omega / \beta_{RA} = 1{,}000$ and $\beta_{RA}/\beta_A = 10$, while varying R_T to modulate the effective molar ratio $R_T^{eff} = R_T / \frac{\alpha_A \Omega}{\beta_{RA}}$. Notably, when the degradation rates of activators alone and in the complex are identical (i.e., $\beta_A = \beta_{RA}$), AT follows a simple Poisson distribution. In contrast, when the repressor promotes activator degradation by binding (i.e., $\beta_A < \beta_{RA}$), the stationary distribution of A_T deviates from Poisson. (e) With the calculated non-Poisson distribution of A_T ($\pi(A_T; R_T, \frac{\alpha_A \Omega}{\beta_{RA}}, \frac{\beta_{RA}}{\beta_A})$), the total mean of M is given by $E[M] = E_\pi\left[\frac{\alpha}{\beta} TA(A_T)\right] = \frac{\alpha}{\beta} \int TA(A_T) \pi\left(A_T; R_T, \frac{\alpha_A \Omega}{\beta_{RA}}, \frac{\beta_{RA}}{\beta_A}\right) dA_T$. Because the transcriptional activity $TA(A_T)$ was set to be identical across all indirect repression models, their total mean mRNA numbers—and thus their effective transcriptional activities under activator fluctuation (i.e., the total mean multiplied by β/α)—are also identical across models: sole sequestration (red), combined sequestration and blocking (blue), and combined sequestration, blocking, and displacement (yellow). (f) Similarly, the total variance of the mRNA copy number can be calculated through the law of the total variance, $Var[M] = \frac{\alpha}{\beta} \int (TA(A_T) - E[M])^2 \pi\left(A_T; R_T, \frac{\alpha_A \Omega}{\beta_{RA}}, \frac{\beta_{RA}}{\beta_A}\right) dR_T +$

$\frac{\alpha}{\beta}\int TA(A_T)FF(A_T)\pi\left(A_T; R_T, \frac{\alpha_A\Omega}{\beta_{RA}}, \frac{\beta_{RA}}{\beta_A}\right)dA_T$. Because the transcriptional activity $TA(A_T)$ was set to be identical across all indirect repression models, the first term is also the same among models. In contrast, the second term differs due to variations in the Fano factor $FF(A_T)$. Consequently, consistent with its lowest Fano factor $FF(A_T)$, the model combining sequestration, blocking, and displacement exhibits the smallest overall mRNA variance, and thus the lowest overall mRNA Fano factor, demonstrating its robustness even under fluctuations in activator copy number.

9. In this work, the authors seem to suggest that cooperative binding to multiple sites has no performance advantage compared to the indirect mechanisms in terms of noise reduction and ultrasensitivity maintenance. Given that many transcription repressors do bind to DNA at multiple sites, I suggest that the authors discuss why this mechanism is still positively selected in evolution.

[Response] We appreciate the reviewer's insightful comment. As the reviewer pointed out, we did not sufficiently discuss the biological rationale for why many systems employ direct cooperative binding rather than indirect repression mechanisms. While incorporating multiple indirect repression mechanisms can provide performance advantages—such as noise resilience and ultrasensitivity—these mechanisms require additional proteins, namely activators, whereas cooperative binding relies solely on repressors. The synthesis and maintenance of extra proteins consume limited cellular resources, potentially constraining the production of other essential proteins and impairing proper cellular function [R10, R11, R12]. This resource burden might in part explain why biological systems with finite resources appear to favor repression strategies that achieve essential regulatory functions with higher resource efficiency.

Page 12 Line 276:

Although multiple indirect repression mechanisms may help improve population-level coherence and enhance noise resilience, these advantages could come at a cost at the single-cell level, particularly when compared with direct repressor–DNA binding. Specifically, indirect repression mechanisms

inherently require the production of additional proteins (i.e., activators), thereby imposing higher energetic and biosynthetic demands on the cell. The synthesis and maintenance of these additional proteins consume substantial biosynthetic resources, and they can limit the cell's capability to produce other essential proteins, which the cell requires to function properly [60-62]. As a result, it is plausible that natural selection often favors simpler regulatory strategies such as direct repressor-DNA binding, which provide efficient transcriptional control with lower biosynthetic cost, even if this comes at the cost of reduced noise resilience and ultrasensitivity. This may explain why many biological systems have evolved to select a direct repressor-DNA binding mechanism [63]. Overall, this trade-off between functional performance and resource limitation may represent a fundamental design principle in transcriptional regulation, and investigating how cells balance these competing demands offers a promising avenue for future research.

Indeed, recent studies have shown that consuming resources, i.e., energy, can enhance functional performance, including ultrasensitivity and noise-resilience, in biological oscillators. For example, we show found that, without energy consumption (i.e., under the detailed balance condition $\frac{K_a K_b}{K_s K_d} = 1$) [21], simply combining indirect repression mechanisms can enhance the noise robustness of biological oscillators (Fig. 4).

10. Multisite binding without cooperativity can induce ultrasensitive responses when it is combined with other mechanisms, such as differential degradation rates from different complexes (doi: 10.1083/jcb.200308060, 10.15252/msb.20209945). These mechanisms may occur at protein or RNA levels, and some of them were shown to reduce noise. I suggest that the authors discuss these alternative mechanisms at different levels that may complement transcriptional regulations that the authors have focused on.

[Response] We sincerely appreciate the reviewer's insightful comment. Although our study focuses on multiple transcriptional repression mechanisms, we agree that various transcriptional mechanisms can

also generate ultrasensitive responses when coupled with other regulatory processes. As the reviewer stated, multisite binding without cooperativity—when combined with mechanisms such as differential degradation or modification rates of protein or RNA complexes—can also induce ultrasensitive response. While our current framework specifically addresses transcriptional repression at the gene-regulation level, we acknowledge that similar principles may extend to post-transcriptional or post-translational regulation, and how such mechanisms influence transcriptional noise remains an open question. To reflect this point, we have revised the Discussion section as follows:

Page 13 Line 305:

Beyond transcriptional repression, several non-transcriptional mechanisms have also been shown to generate ultrasensitive responses in biological systems. For instance, multisite binding without cooperativity can produce ultrasensitivity when coupled with other processes such as differential degradation or modification rates among molecular complexes [66, 67]. Similarly, post-transcriptional regulation through microRNA-mediated repression or RNA-binding proteins can give rise to threshold-like responses and tune gene expression sensitivity [68, 69]. At the post-translational level, protein modification cycles—such as phosphorylation–dephosphorylation cascades or ubiquitination [15, 16, 19] amplify response sensitivity and fine-tune the dynamic range through mechanisms such as enzyme saturation [2, 70]. Therefore, investigating how these non-transcriptional mechanisms operating at translational, post-transcriptional, and post-translational levels influence transcriptional noise would be an interesting direction for future research.

Reviewer #2: The paper by Jeong et al studies the effect of ultra-sensitive transcriptional responses in transcriptional noise. Authors build on their recent papers (references 18 and 19) and explore additional parameter regimes that are physiologically relevant and show that combination of indirect repression mechanisms of sequestration, blocking and displacement can achieve ulrasensitive responses that are associated to small transcriptional noise quantified by Fano factors. This paper is overall well-written and the results are ok. But, I have some concerns about the novelty and significance of the results

presented.

Major comments:

- The advance over the resent work by authors (references 18, 19) seem minimal as it seems mostly an exploration of the new parameter regime. Also, the oscillation example and relevance to some biological systems (Figure 4) and the multiple target case (Fig. 5) is new. I invite the authors to make a better case on the advance developed in this paper.

[Response] We thank the reviewer for this valuable comment. This suggestion made us realize that our manuscript did not sufficiently emphasize how it advances beyond our previous work. In our previous works (Refs. 18, 19), we showed that only incorporating multiple repression mechanisms can yield sensitive transcriptional switches while simultaneously maintaining robustness to noise. However, the present work reveals that this conclusion was incomplete. Specifically, we now show that, regardless of the repression mechanisms, increased activator abundance or faster binding and unbinding kinetics between genes and transcription factors can inherently confer noise robustness. More importantly, our new analysis demonstrates that under biologically realistic conditions — i.e., with physiologically plausible activator concentrations and reaction rates — robustness to noise *can only be achieved* when all three repression mechanisms operate together. Thus, our earlier conclusion remains valid, but only within the biologically relevant parameter space, which we did not fully consider previously due to the simplifying parameter scaling adopted in that study. While we highlighted these results in the original manuscript, we did not explicitly acknowledge the incompleteness of our previous studies. To address this point, we have revised the Introduction, Results, and Discussion sections to make this clear, as follows:

Page 4 Line 82:

Our results show that, *regardless of the specific repression mechanism, indirect repression can reduce noise when DNA binding is fast and activators are abundant, indicating that previous conclusions [22]*

were incomplete. However, only the full combination of all three mechanisms consistently achieves low noise levels and high ultrasensitivity within physiologically plausible constraints.

Page 7 Line 159:

Next, we investigate whether the noise levels can be further decreased as binding and unbinding rates become faster as shown in cooperative binding. Indeed, as binding rates become faster relative to mRNA half-life, the Fano factor decreases in all three models. However, within the biologically relevant range of binding rate and mRNA half-life, the Fano factor still remains high, with a minimum of about 4 (*Table 2* and Fig. 2d, top row). In this case, we used the number of activators $\mathrm{A_T} = 10^3$. *Since the biologically relevant copy number of activators is typically within the range of* $A_T = 10^3 \sim 10^5$ *yoctomole [23], we next tested whether increasing* A_T *could further suppress noise. Indeed, higher* A_T *shifted the low noise region towards the biologically realistic parameter space without altering* k_f *or the mRNA half-life (Fig. 2d, middle row). Notably, when* A_T *was increased to* 10^5*, in combination with faster binding rates and longer mRNA half-lives, the Fano factor approached ~1 in all three models (Fig. 2d, bottom row).*

To further investigate noise levels under conditions of higher ultrasensitivity, we adjusted the dissociation constants of the indirect repression mechanisms to produce transcriptional activity with a Hill coefficient of 50 (Fig. 3a). This elevated ultrasensitivity is accompanied by increased transcriptional noise (Fig. 2c and 3b). *This increase amplifies the differences among the models, which originate from stochastic transitions of DNA states (Supplementary Fig. 3) [22]. Furthermore, such attenuation of transcriptional noise—while maintaining comparable levels of ultrasensitivity through the addition of repression mechanisms—persists even when the numbers of activators and repressors fluctuate due to processes such as repressor birth–death dynamics and repressor-induced activator degradation (Supplementary Fig. 4). This effect is robust across a broad parameter range, and stronger repression further enhances noise suppression (Supplementary Fig. 5). Accordingly, under biologically relevant conditions, only the combination of sequestration, blocking, and displacement exhibits a low*

Fano factor ranging from 1 to 20—consistent with the experimentally observed range in mammalian cells—and is uniquely capable of reducing it to values close to 1 [37] (Fig. 3c).

Page 11 Line 251:

However, those studies did not account for the timescales of DNA-binding dynamics (i.e., the binding and unbinding rates between DNA and transcriptional factors). *Here, we show that this omission can be misleading:* when DNA binding occurs on a faster timescale than mRNA-related processes (i.e., transcription and degradation), ultrasensitivity and noise robustness can be simultaneously achieved in the resulting transcriptional switch (Fig. 1b–e, 2b, and d)*, regardless of the underlying repression mechanism*. We further show that increasing the number of activators can also promote such ultrasensitive behavior (Fig. 2b–d).

In addition, as the reviewer points out, the oscillatory system example (Fig. 4) and the multiple target case (Fig. 5) provide further novelties of this study. In addition, to address the reviewer's next comment, we have also added new simulations in which the number of repressors fluctuates. Finally, in response to other reviewer comments, we have incorporated additional analyses such as noise in cooperative activation, effect of fluctuation of activator numbers, and validity check of QSSA, which further strengthen the contribution of the present work.

- If I understand correctly, all the results both for the direct and indirect repressor assumes that the level of repressor is constant. But, in reality repressor is another protein and is being expressed stochastically and noise in the repressor can cascade through the transcriptional regulation to the downstream gene expression. This can be considered as a form of extrinsic noise. Is the proposed indirect repression mechanism also robust to this kind of noise?

[Response] We sincerely appreciate the reviewer's suggestion to consider stochastically expressed repressors. As the reviewer noted, repressors are often expressed stochastically; for example, when their copy number follows simple birth–death dynamics, it obeys a Poisson distribution (Supplementary Fig.

4a). To address this point, we numerically computed the Fano factor of the mRNA copy number when the number of repressors follows a distribution rather than remaining constant. As we had already derived expressions for the transcriptional activity and the Fano factor under a fixed number of repressors (Table 2), the overall mean, variance, and Fano factor of mRNA numbers can be obtained using the laws of total expectation and total variance.

First, according to the law of total mean, the overall mean of the mRNA number is given by $E[M] = E_\pi[E[M|R_T]]$, where M is the mRNA number, R_T is the total number of repressors, and π denotes the probability density function of distribution that R_T follows. For given R_T, the conditional mean of M (i.e., $E[M|R_T]$) can be calculated as the product of the production-to-degradation rate ratio (α/β) and the transcriptional activity $TA(R_T)$ from Table 2; that is, $E[M|R_T] = \frac{\alpha}{\beta}TA(R_T)$. Hence, the overall mean of the mRNA number is given by

$$E[M] = E_\pi\left[\frac{\alpha}{\beta}TA(R_T)\right] = \frac{\alpha}{\beta}\int TA(R_T)\pi(R_T)dR_T \ .$$

Because the transcriptional activity was set to be identical across all indirect repression models (Fig. 3a), their overall mean mRNA levels are also identical, regardless of π (Supplementary Fig 4b).

Next, according to the law of total variance, the overall variance of the mRNA number is given by

$Var[M] = Var_\pi[E[M|R_T\ ]] + E_\pi[Var[M|R_T]]$. For given R_T, the conditional variance of M (i.e., $Var[M|R_T]$) can be expressed as the product of $E[M|R_T]$ and the Fano factor $FF(R_T)$ from Table 2; that is, $Var[M|R_T] = \frac{\alpha}{\beta}TA(R_T)FF(R_T)$. Hence, the overall variance of the mRNA number is given by

$$Var[M] = Var_\pi\left[\frac{\alpha}{\beta}TA(R_T)\right] + E_\pi\left[\frac{\alpha}{\beta}TA(R_T)FF(R_T)\right]$$

$$= \frac{\alpha}{\beta}\int (TA(R_T) - E[M])^2\pi(R_T)dR_T + \frac{\alpha}{\beta}\int TA(R_T)FF(R_T)\pi(R_T)dR_T.$$

Because of identical transcriptional activities across all indirect repression models (Fig. 3a), the first term $Var_\pi\left[\frac{\alpha}{\beta}TA(R_T)\right]$ is the same for all. In contrast, their Fano factors—and thus the second term

$E_\pi\left[\frac{\alpha}{\beta}TA(R_T)FF(R_T)\right]$—differ among models (Fig. 3b). Consequently, consistent with its lowest Fano factor $FF(R_T)$, the model combining sequestration, blocking, and displacement exhibits the smallest overall mRNA variance, and thus the smallest overall mRNA Fano factor (Supplementary Fig. 4c). These results demonstrate that the robustness of the proposed models is preserved, even under fluctuations in repressor copy number, regardless of the distribution that R_T follows. These findings are now presented in Supplementary Fig. 4, and the Results section has been revised as follows:

Page 8 Line 169:

To further investigate noise levels under conditions of higher ultrasensitivity, we adjusted the dissociation constants of the indirect repression mechanisms to produce transcriptional activity with a Hill coefficient of 50 (Fig. 3a). This elevated ultrasensitivity is accompanied by increased transcriptional noise (Figs. 2c and 3b). *This increase amplifies the differences among the models, which originate from stochastic transitions of DNA states (Supplementary Fig. 3) [22]. Furthermore, such attenuation of transcriptional noise—while maintaining comparable levels of ultrasensitivity through the addition of repression mechanisms—persists even when the numbers of activators and repressors fluctuate due to processes such as repressor birth–death dynamics and repressor-induced activator degradation (Supplementary Fig. 4). This effect is robust across a broad parameter range, and stronger repression further enhances noise suppression (Supplementary Fig. 5). Accordingly, under biologically relevant conditions, only the combination of sequestration, blocking, and displacement exhibits a low Fano factor ranging from 1 to 20—consistent with the experimentally observed range in mammalian cells—and is uniquely capable of reducing it to values close to 1 [37] (Fig. 3c).*

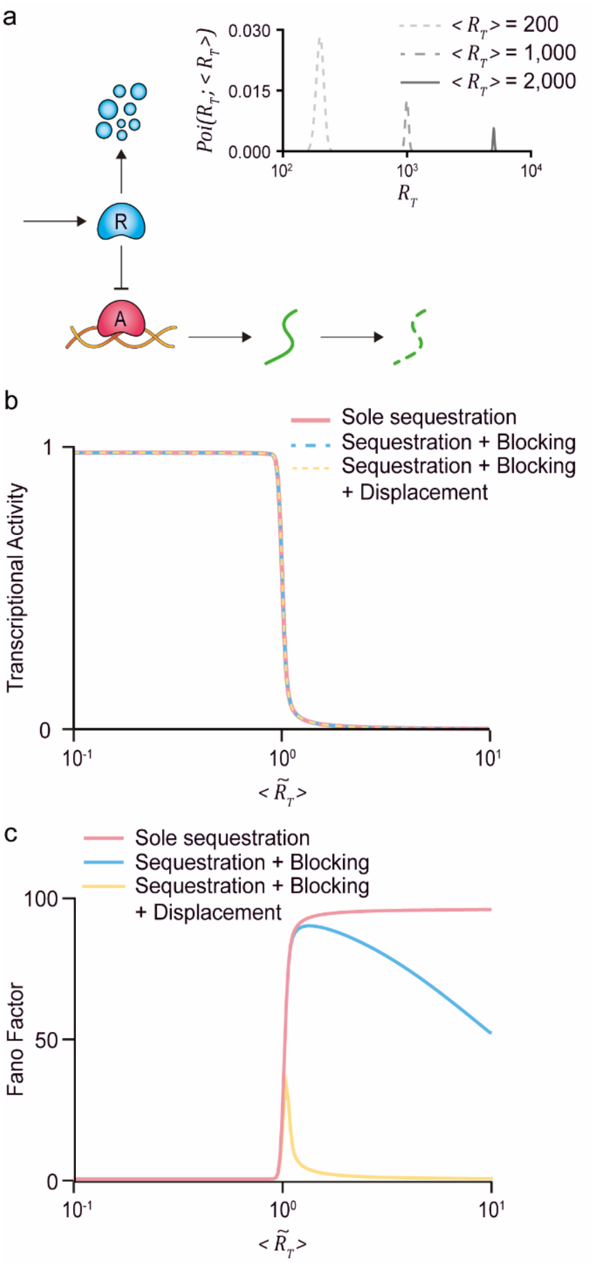

Supplementary Figure 4. Despite fluctuations in repressor or activator copy number, the combined sequestration, blocking, and displacement model maintains robustness of noise reduction.

(a) When a repressor is regulated by a simple birth–death process, its copy number (R_T) follows a Poisson distribution with the mean of $< R_T >$. (b) In this case, the total mean of the mRNA copy number (M) can be calculated through the law of the total mean, $E[M] = E_\pi[E[M|R_T]]$, where denotes the probability mass function of Poisson distribution with the mean of $< R_T >$ and $E[M|R_T]$ is the conditional mean of M for a given R_T. As $E_\pi[E[M|R_T]]$ can be calculated as the product of the

production-to-degradation rate ratio (α/β) *and the transcriptional activity* $TA(R_T)$ *in Table 2 (i.e.,* $E[M|R_T] = \frac{\alpha}{\beta}TA(R_T)$*), the total mean of* M *becomes* $E[M] = E_\pi\left[\frac{\alpha}{\beta}TA(R_T)\right] = \frac{\alpha}{\beta}\int TA(R_T)\pi(R_T;< R_T >)dR_T$*. Because the transcriptional activity* $TA(R_T)$ *was set to be identical across all indirect repression models, their total mean mRNA numbers—and thus their effective transcriptional activities under repressor fluctuation (i.e., the total mean multiplied by* β/α*)—are also identical across models: sole sequestration (red), combined sequestration and blocking (blue), and combined sequestration, blocking, and displacement (yellow). (c) Similarly, the total variance of the mRNA copy number can be calculated through the law of the total variance,* $Var[M] = Var_\pi[E[M|R_T]] + E_\pi[Var[M|R_T]]$*, where* $Var[M|R_T]$ *is the conditional variance of* M *for a given* R_T*. As* $Var[M|R_T]$ *can be calculated as the product of the product of* $E[M|R_T]$ *and the Fano factor* $FF(R_T)$ *in Table 2 (i.e.,* $\frac{\alpha}{\beta}TA(R_T)FF(R_T)$*), the total variance of* M *becomes* $Var[M] = \frac{\alpha}{\beta}\int (TA(R_T) - E[M])^2\pi(R_T;< R_T >)dR_T + \frac{\alpha}{\beta}\int TA(R_T)FF(R_T)\pi(R_T;< R_T >)dR_T$*. Because the transcriptional activity* $TA(R_T)$ *was set to be identical across all indirect repression models, the first term is also the same among models. In contrast, the second term differs due to variations in the Fano factor* $FF(R_T)$*. Consequently, consistent with its lowest Fano factor* $FF(R_T)$*, the model combining sequestration, blocking, and displacement exhibits the smallest overall mRNA variance, and thus the lowest overall mRNA Fano factor, demonstrating its robustness even under fluctuations in repressor copy number.*

- I was not completely clear what is done exactly with regards to the results in Figure 5. If the ratio of repressors to number of genes being regulated is close to 1 I would imagine the regulation becomes very noisy. I would like some more explanation about this. Also, this could be better presented as part of results rather than discussion.

[Response] We thank the reviewer for this insightful comment. We agree that the rationale for studying multiple target genes and the interpretation of the results were not sufficiently clear in the original

manuscript. In response to the reviewer's suggestion, we have moved the relevant paragraph from the Discussion section to the Results section and clarified the corresponding motivation and details.

Previous studies have suggested that regulatory systems often exhibit increased noise when multiple genes share limited activators, as competition among targets can amplify fluctuations and destabilize expression levels. This is why we specifically examined the case in which the ratio of repressors to regulated genes is close to 1 — a scenario that is generally expected to lead to a high level of noise, as the reviewer pointed out.

Surprisingly, our simulations revealed that the proposed triple repression mechanism (sequestration, blocking, and displacement) maintained noise robustness even in this challenging regime. Specifically, in the absence of repressors, multiple target genes indirectly suppress each other by competing for activators, effectively acting as mutual repressors through sequestration. This competition functions as an additional layer of sequestration even within the combined sequestration, blocking, and displacement model. As a result, the noise level increased and became comparable to that of the sole sequestration model, when the number of target genes is similar to or greater than that of activators (red circle in Fig. 5), consistent with both previous findings and the reviewer's expectation. However, as the number of repressors increased, direct repression became dominant, and the Fano factor converged to that observed in the single-target case with multiple repressions. These results indicate that, even when the ratio of repressors to target genes is close to one, the proposed triple repression mechanism can maintain noise robustness— a nontrivial finding that challenges the common view that multi-target regulation necessarily amplifies noise.

To clarify this point, we have (i) revised the Results section to include this rationale and interpretation, and (ii) updated Figure 5 and the accompanying text to more explicitly describe how these simulations were performed and why they are biologically and theoretically significant, as follows:

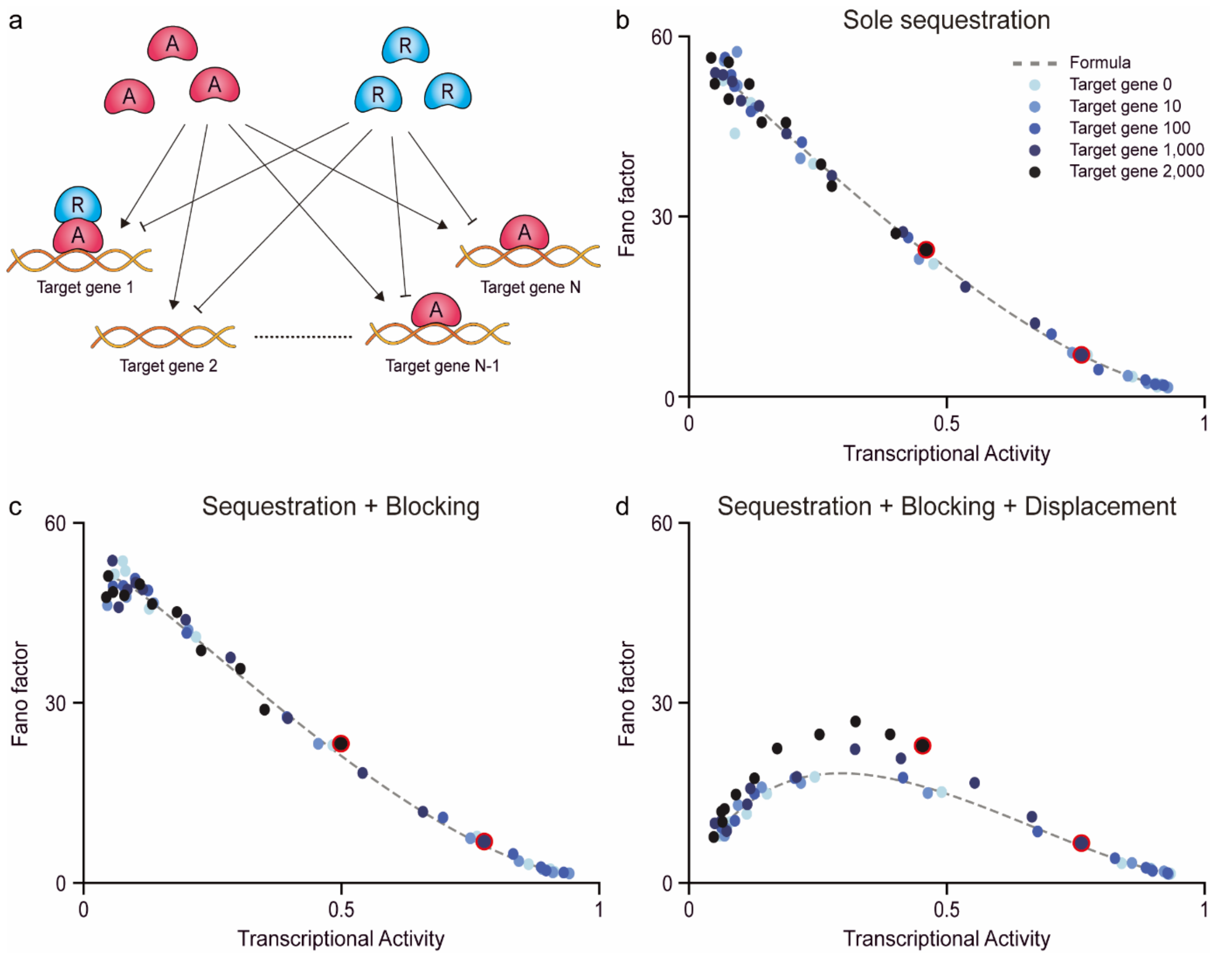

Page 9 Line 213:

The combination of indirect repressions preserves noise robustness even under multi-target gene regulation

We analyzed models in which 1,000 to 100,000 transcriptional activators regulate a single target gene (Fig. 2a). However, in many biological systems employing multiple repression mechanisms—such as the circadian clock, NF-κB oscillator, and p53-Mdm2 oscillator (Fig. 4a–c)—transcriptional activators often regulate hundreds to thousands of genes simultaneously (Fig. 5a). For example, in the mammalian circadian clock, a limited pool of BMAL1 proteins regulates approximately 3,400 genes [50]; p53 controls around 3,700 target genes [51]; and NF-κB targets several hundred genes [52].

Previous studies have suggested that such shared regulation can increase noise, as multiple target genes compete for a limited pool of transcriptional activators [53, 54]. To examine whether our

proposed mechanisms can overcome this effect, we extended each indirect repression model to include varying numbers of target genes, with regulation mediated through the corresponding repression mechanisms (Fig. 5a and Table 1). Notably, increasing the number of target genes has a similar effect as increasing the number of repressors, as it reduces transcriptional activity for the same number of repressors. Thus, for *fair* comparisons, we evaluated the noise level of each model at equivalent levels of transcriptional activity rather than with equal numbers of repressors *(Fig. 5b-d)*.

Surprisingly, we found that the combination of three indirect repressions preserves noise robustness even under multi-target gene regulation. When the number of the target genes was smaller than that of activators (i.e., 1,000), the transcriptional noise of individual genes remained largely unaffected (Fig. 5b–d, Target gene 0, 10, and 100). Specifically, regardless of repression via the sole sequestration (Fig. 5b), the combination of sequestration, and blocking (Fig. 5c), or the combination of sequestration, blocking, and displacement (Fig. 5d), the Fano factor for multiple targets closely matched those of single-target regulation (gray dashed lines). In contrast, when the number of target genes exceeded that of activators (Fig. 5b-d, Target gene 1,000, and 2,000), the Fano factor slightly deviated from the single-target case in the full combination model (Fig. 5d), but not in the others (Fig. 5b and c). This deviation arose because, in the absence of repressors, multiple target genes indirectly suppressed each other by depleting the available activator pool (Fig. 5d, red-circled points). This competition effectively acts as an additional layer of sequestration, even in the combined repression model, increasing the noise to a level comparable to that of the sole sequestration model. However, as the number of repressors increased (i.e., as the transcriptional activity decreased), the multiple repression effects became dominant. Consequently, the Fano factor converged to that of the single-target case with multiple repressions (Fig. 5d). These results indicate that, contrary to the conventional expectation that multi-target regulation amplifies transcriptional noise, the combined sequestration, blocking, and displacement mechanisms can buffer against such fluctuations, enabling a finite pool of activators to robustly regulate large gene networks.

Minor comments:

- Why is Figure 1a is not showing all possible states? I assumed that all states are allowed in the model. If this is just for illustration purposes should be explained in the caption.

[Response] We thank the reviewer for pointing this out. In Figure 1a, we presented only a subset of states for clarity and conciseness. Following the reviewer's suggestion, we have revised Figure 1a to now include all possible states in the model, ensuring consistency between the illustration and the full mathematical framework as follows:

a

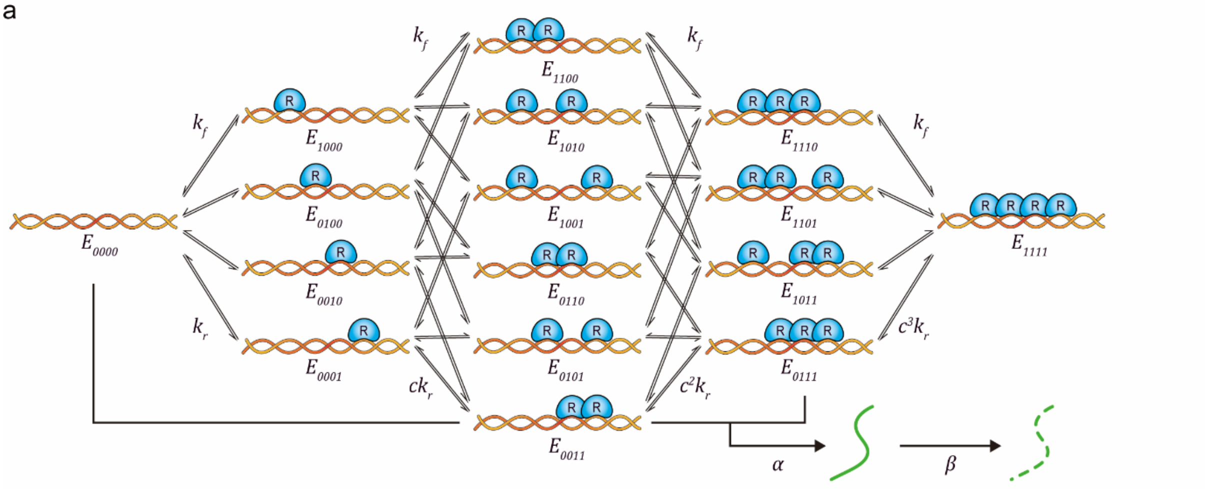

- Table 1 bottom of page 10 in second column last line says "with the" seems like a word is missing.

[Response] We thank the reviewer for catching this typo. The phrase "with the" has been corrected to "with the blocking" in the revised manuscript.

- No code for the simulations has been included.

[Response] We apologize for this oversight. We have now included the codes for all simulations, including those newly added during the review process, as well as those originally implemented during manuscript preparation. The codes for all simulations are available at GitHub now (https://github.com/Mathbiomed/Ultrasensitive-gene-switch).

Reviewer #3: Ultrasensitive transcriptional response is a fundamental phenomenon in gene expression and gene regulation, playing a key role in shaping diverse biological behaviors. In this manuscript, the authors address an important and timely question: how can cells maintain low transcriptional noise while achieving highly ultrasensitive transcriptional responses under physiologically realistic conditions? Through careful modeling and analysis, the authors demonstrate that only the full combination of three indirect repression mechanisms—sequestration, blocking, and displacement—can consistently achieve both goals. The manuscript is well written, and the results are compelling and potentially impactful. I recommend publication after the following comments are adequately addressed.

Major points

1. The authors derive explicit analytical expressions for the transcriptional activities and Fano factors for three different models. It would strengthen the manuscript if the authors could further analyze these expressions and provide theoretical insights into why the combination of all three indirect repression mechanisms leads to low transcriptional noise, whereas combinations of only two do not. A mathematical or mechanistic explanation would significantly enhance the readers' understanding.

[Response] We thank the reviewer for this insightful comment. As the reviewer suggested, to provide theoretical insights, we first attempted a direct analytical comparison of the Fano factor expressions across the three indirect repression models. However, this approach was not tractable due to the presence of multiple nonlinear and squared terms. Instead, to identify the mechanistic explanation of how all three indirect repression mechanisms work together to attenuate noise, we analyzed the distributions of mRNA numbers for each model (Supplementary Fig. 3a–c) using parameter sets corresponding to a molar ratio of repressors and activators equal to 5 ($\widetilde{R_T} = 5$). Under this condition, strong suppression would normally be expected. However, both the sole sequestration model and the combined sequestration and blocking model exhibited bimodal mRNA distributions with peaks at both

low and high mRNA numbers (Supplementary Fig. 3a–b), indicating that an activated DNA state persisted even in the presence of many repressors. In contrast, when displacement was additionally incorporated (the combined sequestration, blocking, and displacement model), the mRNA distribution became unimodal (Supplementary Fig. 3c), reflecting consistent suppression of transcription. Consequently, this model showed the lowest variability, consistent with the reduced Fano factor observed in Figs. 2c and 3b. Even at a large molar ratio (i.e., when repressors outnumber activators), the sequestered activator can stochastically unbind from the repressor and rebind to DNA to form E_A (Supplementary Fig. 3d–f(i), at time 0). In the sole sequestration model, once E_A is formed, it persists for a long time (Supplementary Fig. 3d(i)) until the DNA-bound activator eventually unbinds from DNA itself and returns to the E_F state (Supplementary Fig. 3(ii)). This long-lasting E_A state causes a continuous increase in the mRNA copy number (Supplementary Fig. 3g), resulting in a bimodal mRNA distribution (Supplementary Fig. 3a) and thus a high Fano factor. In contrast, in the combined sequestration and blocking model, once E_A is formed (Supplementary Fig. 3e(i)), it rapidly transitions to the repressed state E_R via blocking (Supplementary Fig. 3e(ii)). Conversely, E_R can also revert to E_A when the repressor dissociates from the repressor–activator–DNA complex. These frequent transitions between the E_A and E_R states continue until the activator eventually unbinds from DNA to form the E_F state (Supplementary Fig. 3e(iii)), similar to the sole sequestration model. Such frequent blocking events delay the accumulation of mRNA (Supplementary Fig. 3h), resulting in two peaks that are closer together in the mRNA distribution (Supplementary Fig. 3b) compared to the sole sequestration model (Supplementary Fig. 3a). In the combined sequestration, blocking, and displacement model, once E_A is formed (Supplementary Fig. 3f(i)), it quickly transitions to the repressed state E_R (Supplementary Fig. 3f(ii)) and then to the free E_F state through displacement (Supplementary Fig. 3f(iii)). This rapid displacement further suppresses the persistence of the E_A state, resulting in a unimodal mRNA distribution (Supplementary Fig. 3c) and thus a low Fano factor. In light of these observations, we have revised the Results section and included the new analyses in Supplementary Fig. 3, as follows:

Page 8 Line 169: *To further investigate noise levels under conditions of higher ultrasensitivity, we adjusted the dissociation constants of the indirect repression mechanisms to produce transcriptional activity with a Hill coefficient of 50 (Fig. 3a).* This elevated ultrasensitivity is accompanied by increased transcriptional noise (Figs. 2c and 3b). *This increase amplifies the differences among the models, which originate from stochastic transitions of DNA states (Supplementary Fig. 3) [22].*

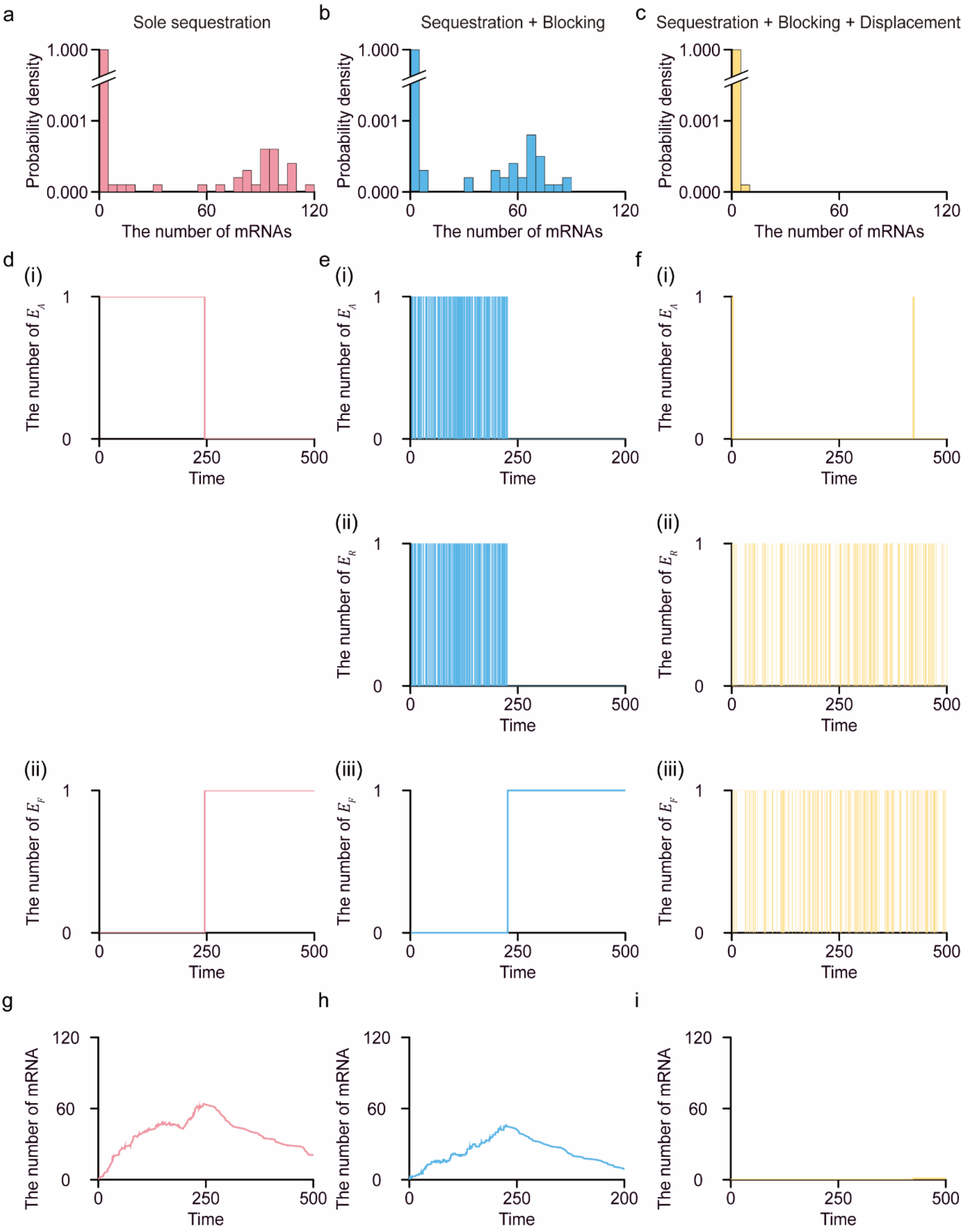

Supplementary Figure 3. Simulated time-series of DNA states and mRNA copy numbers, and stationary distribution of mRNA copy numbers across the three repression models.

(a–c) Stationary distributions of mRNA copy numbers for the sole sequestration (a), combined sequestration and blocking (b), and combined sequestration, blocking, and displacement models (c), simulated using the parameters in Fig. 3 at $\widetilde{R_T} = 5$. *Despite the large number of repressors compared to the activators, both the sole sequestration model and the combined sequestration and blocking model exhibited bimodal mRNA distributions, with peaks at both low and high mRNA numbers, indicating that the activated DNA state persisted even in the presence of many repressors. In contrast, incorporation of displacement rendered the mRNA distribution unimodal, reflecting more consistent transcriptional repression. (d-i) Even under a large excess of repressors over activators, the sequestered activator can stochastically dissociate and rebind to DNA to form the active complex* E_A *(d–f(i), at time 0). In the sole sequestration model (d), once the activator binds to DNA to form* E_A *(d(i)), it remains bound for a long duration before dissociating into* E_F *(d(ii)). This leads to continuous mRNA accumulation (g), and thus a bimodal mRNA distribution with a high Fano factor. In the combined sequestration and blocking model (e),* E_A *rapidly transitions to the repressed state* E_R *via blocking (e(i) and (ii)), and frequently interconverts between* E_A *and* E_R *until the activator dissociates from DNA to form* E_F *(e(iii)). These frequent blocking events delay mRNA accumulation (h), and reduce the separation between the two peaks in the mRNA distribution, resulting in a lower Fano factor than in the sole sequestration model. In contrast, in the combined sequestration, blocking, and displacement model (f),* E_A *rapidly transitions to* E_R *(f(i) and f(ii)) and displacement accelerates the transition from* E_R *to* E_F *(f(iii)), thereby preventing mRNA accumulation (i) and producing a unimodal mRNA distribution with a low Fano factor.*

Furthermore, because a direct analytical comparison of the Fano factor expressions was not feasible for explaining the progressive noise reduction with the addition of indirect repression mechanisms, we next examined this trend across a broad parameter range. in a more continuous and systematic manner. To this end, we tuned the dissociation constants K_a and K_s, and evaluated the sensitivity of transcriptional activity and the overall noise level, quantified by the Hill coefficient (Supplementary Fig. 5a) and the area under the Fano factor curve (AUC; Supplementary Fig. 5b), respectively. In the

sole sequestration model, strong sequestration (i.e., $K_s \ll K_a$) was required to generate ultrasensitivity (Supplementary Fig. 5a(i)); however, this parameter regime also exhibited a high overall noise level (Supplementary Fig. 5b(i)), indicating that ultrasensitivity is inevitably accompanied by increased noise. When blocking was added with $K_b = 10^{-3}$, ultrasensitivity was maintained (Supplementary Fig. 5a(ii)), while the overall noise level was mitigated (Supplementary Fig. 5b(ii)), indicating that the blocking is capable of dampening fluctuations without loss of ultrasensitivity. Strengthening the blocking further ($K_b = 10^{-4}$) reduced noise even more, while maintaining comparable ultrasensitivity. Regardless of blocking strength, the inclusion of displacement preserved the overall transcriptional activity with $K_d = K_a K_b / K_s$ (Supplementary Fig. 5a(iv–v)), while further reducing the noise level (Supplementary Fig. 5b(iv–v)), indicating cooperative noise attenuation among the repression mechanisms. When the displacement strength was increased (i.e., $K_d = 100 K_a K_b / K_s$), transcriptional sensitivity was further enhanced (Supplementary Fig. 5a(vi-vii)), accompanied by a lower overall noise level (Supplementary Fig. 5b(vi-vii)) compared to the weaker displacement case (Supplementary Fig. 5a(iv-v) and 5b(iv-v)). Collectively, the addition of each repression mechanism reduced noise, and as the strength of repression increased, this reduction became more pronounced while ultrasensitivity was either maintained or further amplified. We have now added this result as a new Supplementary Fig. 5 and incorporated it into the revised Results section as follows:

Page 8 Line 169: *To further investigate noise levels under conditions of higher ultrasensitivity, we adjusted the dissociation constants of the indirect repression mechanisms to produce transcriptional activity with a Hill coefficient of 50 (Fig. 3a).* This elevated ultrasensitivity is accompanied by increased transcriptional noise (Figs. 2c and 3b). *This increase amplifies the differences among the models, which originate from stochastic transitions of DNA states (Supplementary Fig. 3) [22]. Furthermore, such attenuation of transcriptional noise—while maintaining comparable levels of ultrasensitivity through the addition of repression mechanisms—persists even when the numbers of activators and repressors fluctuate due to processes such as repressor birth–death dynamics and repressor-induced activator degradation (Supplementary Fig. 4). This effect is robust across a broad parameter range, and stronger repression further enhances noise suppression (Supplementary Fig. 5). Accordingly, under biologically*

relevant conditions, only the combination of sequestration, blocking, and displacement exhibits a low Fano factor ranging from 1 to 20—consistent with the experimentally observed range in mammalian cells—and is uniquely capable of reducing it to values close to 1 [37] (Fig. 3c).

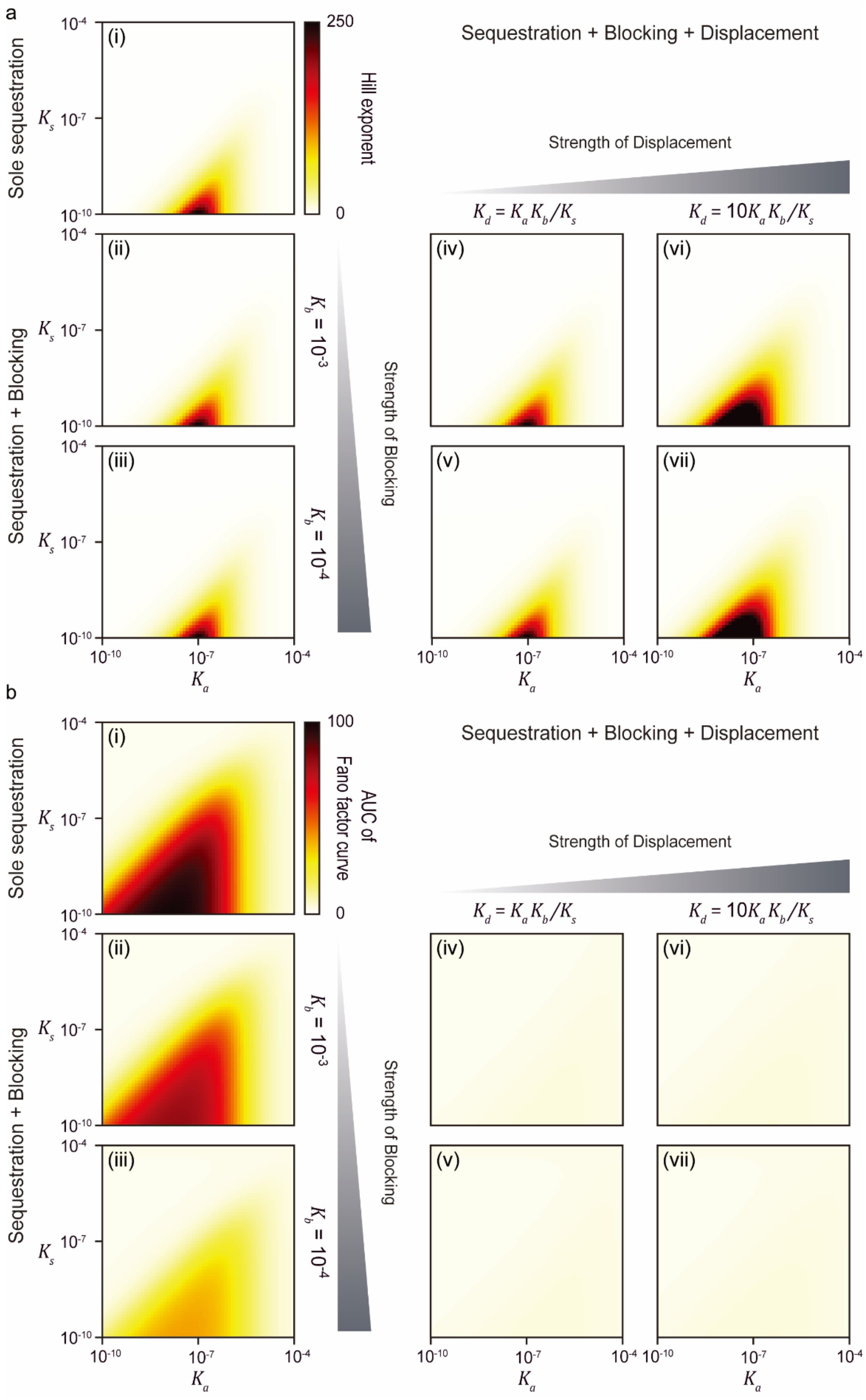

Supplementary Figure 5. Systematic evaluation of the effects of indirect repression mechanisms on ultrasensitivity of transcriptional activity and overall noise level.

(a - b) Heatmaps showing the ultrasensitivity of transcriptional activity and the overall noise level, quantified by the Hill coefficient (a) and the area under the Fano factor curve (AUC) over a range of $\widetilde{R_T}$ *from* 10^{-1} *to* 10^{1} *(b), respectively, by varying the dissociation constants* K_a *and* K_s *in the three indirect repression models. In the sole sequestration model, strong sequestration (i.e.,* $K_s \ll K_a$*) promoted high ultrasensitivity (a(i)) but was accompanied by elevated noise levels (b(i)). Incorporating blocking, with* $K_b = 10^{-3}$*, maintained a similar level of ultrasensitivity as in the sole sequestration case (a(ii)), while reducing the overall noise (b(ii)), indicating that the addition of blocking dampens fluctuations without compromising sensitivity. Further strengthening the blocking (*$K_b = 10^{-4}$*) lowered the noise even more (b(iii)) without loss of ultrasensitivity (a(iii)). Regardless of blocking strength, adding displacement, with its rate set to* $K_d = K_a K_b / K_s$ *to maintain comparable ultrasensitivity (a(iv-v)), led to additional noise reduction (b(iv-v)), demonstrating cumulative noise suppression through cooperative multiple repressions. Moreover, stronger displacement (*$K_d = 10 K_a K_b / K_s$*) produced higher ultrasensitivity (a(vi-vii)) with sustained low noise (b(vi-vii)) compared to the weaker displacement (*$K_d = K_a K_b / K_s$*; a(iv-v) and b(iv-v)). Taken together, the sequential addition and strengthening of repression mechanisms progressively reduced noise while retaining or amplifying ultrasensitivity.*

2. In Figure 1, the authors study ultrasensitivity generated by cooperative binding in the context of transcriptional repression. They show that this mechanism fails to simultaneously maintain low noise and high ultrasensitivity. Would the same conclusion hold if transcription were regulated by an activator protein instead of a repressor? A brief discussion or clarification of this scenario would be helpful for readers interested in activator-based regulation.

[Response] We thank the reviewer for raising this insightful question. The key conclusion in Figure 1—that ultrasensitivity generated by cooperative binding cannot simultaneously achieve low noise and high ultrasensitivity—also holds when considering activator-based regulation. Specifically, Sánchez et al. have shown that cooperativity in a two–binding-site promoter increases noise compared to the non-

cooperative case. To further illustrate this, we constructed a model with four binding sites in which transcription occurs only when all four sites are occupied by activators. Consistent with the repressor-based regulation, we found that ultrasensitive activation is also accompanied by high noise levels. Now, we have newly added this result to the Results section as follows:

Page 5 Line 117:

When k_f and k_r became slow relative to the rates of mRNA production and degradation (i.e., α and β), slow transitions between the active and repressed DNA states resulted in bimodal mRNA stationary distributions (Fig. 1d, top). *Such noise attenuation by fast* k_f *and* k_r *was also observed in an activator model, where transcription occurs only when an activator protein binds cooperatively to all four sites (Supplementary Fig. 1), consistent with Sánchez et al [28].*

Next, we investigate whether the noise level in transcription decreases as the binding and unbinding rates become faster across a broad parameter range.

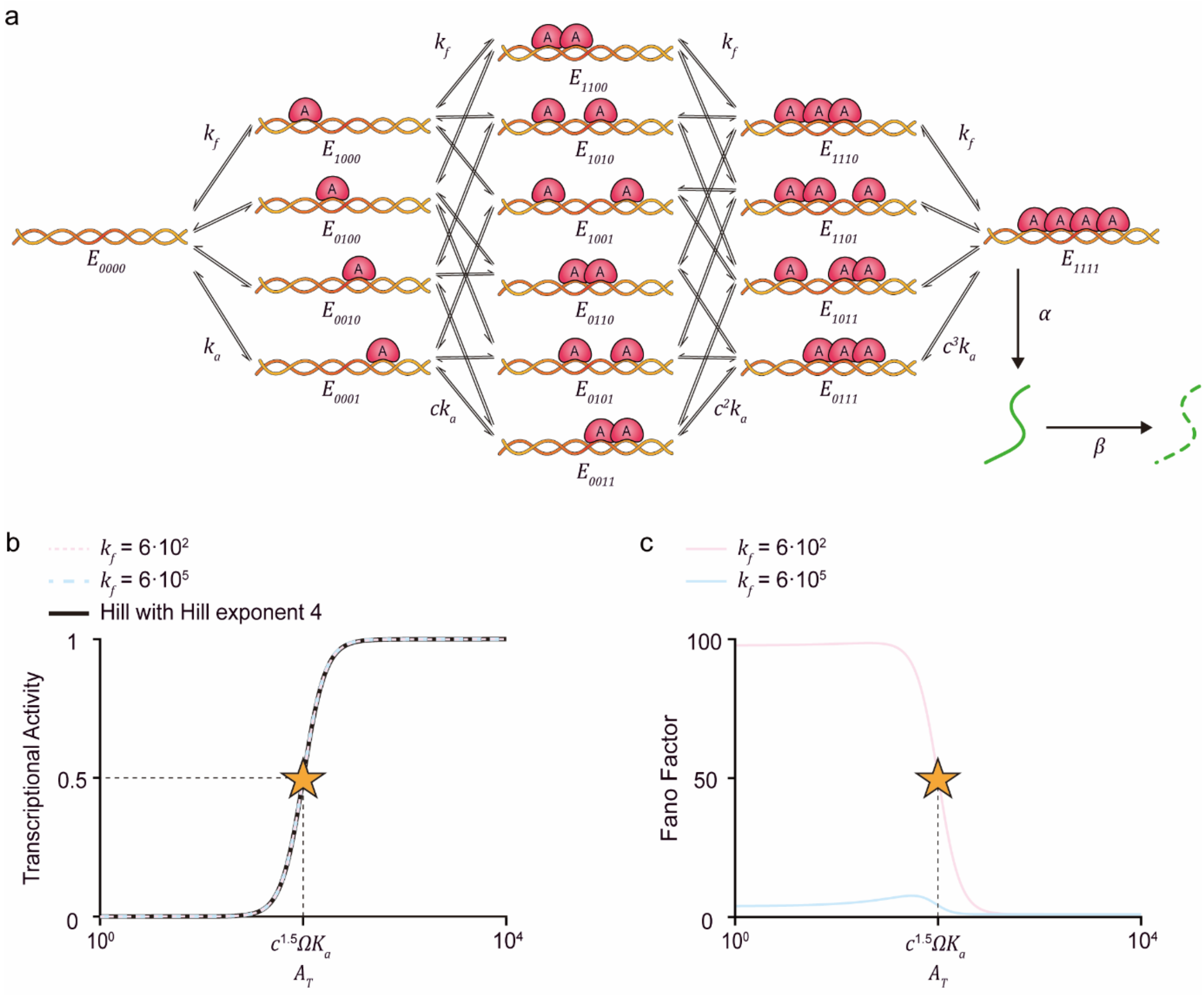

Supplementary Figure 1: The cooperative binding mechanism, in which activator binding activates transcription, is sensitive to noise.

(a) Model diagram of the transcription regulated by the activator proteins (A) binding to four independent sites on the DNA within a cell volume of Ω. Each binding site is occupied by A at a rate k_f. Conversely, A unbinds from DNA at a rate k_a when one site is occupied, with the dissociation constant between A and DNA defined as $K_a = k_a/k_f$. For two, three, or four occupied sites, A unbinds at rates ck_a, c^2k_a and c^3k_a, respectively. Accordingly, when $c < 1$, cooperative binding is present. When all binding sites are occupied, mRNA is produced at a rate α and degrades at a rate β, whereas transcription is inactive if any site remains unoccupied. (b) When $c = 10^{-2}$, transcriptional activity closely resembles the Hill function with a Hill exponent of 4 (black line). Furthermore, transcriptional activity remains consistent with the Hill function regardless of k_f values,

provided K_a is kept constant (red dotted line and blue dashed line). (c) Nevertheless, the overall noise level quantified by the Fano factor of mRNAs shows significant differences based on k_f. Notably, the overall noise levels are reduced during the sensitive response when k_f is faster (red solid line) compared to when it is slower (blue solid line).

3. On page 6 and elsewhere, the authors present ranges for certain key parameters. However, such parameters can vary substantially across different cell types. It would be informative if the authors could specify the biological system or cell type that these parameter ranges are based on. Furthermore, providing a summary of typical Fano factor ranges observed across different cell types would give useful context to interpret the modeling results.

[Response] We appreciate the reviewer's valuable feedback. Most parameters used in this study were obtained from typical mammalian cells, while several rate constants were derived from *in vitro* or *in silico* theoretical studies. Specifically, cell volume (Ω) was referenced from typical mammalian cells, with its value in the range of 10^{-12} to 10^{-11} l [R1]. The mRNA half-life was taken from mouse embryonic stem cells and human whole blood, where their measurements report values ranging from less than 1 hour to up to 16.4 hours [R2, R3]. The concentration of activators (A_T/Ω) in *H. sapiens* cell lines typically lies between $10^{-9}M$ and $10^{-7}M$ [R1]. For the binding rate (k_f), it was theoretically estimated by relating the dissociation constant to the interdependence between binding and unbinding rates, while considering physical and diffusion constraints. These frameworks predict the protein-protein binding rate of $10^6 \sim 10^7 M^{-1}s^{-1}$ [R4]. Indeed, the binding rate between p53 and Mdm2 ($9.2 \pm 0.04 \times 10^6 M^{-1}s^{-1}$) determined from *in silico* molecular dynamics simulations [R5], and that of the transcriptional activator NF-κB binding to ($10^6 M^{-1}s^{-1}$) measured *in vitro* in *E. coli* BL21 (DE3) expressing human NF-κB [R6], both fall within this range. Based on these clarifications, we have added a new table that explicitly specifies the corresponding cell types for each parameter, as follows:

Page 6 Line 125:

However, achieving this requires biologically unrealistic fast binding and unbinding rates. Specifically, with a biologically relevant binding rate $k_f \Omega^{-1} = 6 \times 10^{-6} \sim 6 \times 10^{-5}$ $ymol^{-1}min^{-1}$ *[24, 29, 30]*, where $\Omega = 10^7 aL$ is the volume of the cell [23], and the mRNA half-life is 0.5 ~ 16.4 hours (i.e., 30 ~ 984 min; *Table 2*) [31, 32], the Fano factor is greater than 40 (Fig. 1e, gray box). Taken together, while increasing binding rates can minimize noise, the cooperative binding mechanism remains highly noisy under physiological conditions.

Page 7 Line 161:

However, within the biologically relevant range of binding rate and mRNA half-life, the Fano factor still remains high, with a minimum of about 4 (*Table 2 and* Fig. 2d, top row). In this case, we used the number of activators $A_T = 10^3$.

Page 41 Line 755:

Table 2. The references of parameter values used for model simulations.

Cell type	***Parameter (definition)***	***Reported range***	***References***
In vitro (Cell host: human NF-κB and IκBα expressed in E. coli BL21 DE3); N/A – Theoretical / literature-based study; In silico molecular dynamics simulation	k_f *(binding rate)*	$10^6 \sim 10^7\ M^{-1}s^{-1}$	*Williamson, 2023; Bergqvist et al., 2009; Zou et al., 2020*
Typical mammalian cells	*Ω (cell volume)*	$10^{-12} \sim 10^{-11} l$	*Milo et al., 2016*
Mouse embryonic stem cells, human whole blood	*ln 2 / β (mRNA half-life)*	*0.5 (less than 1 h) ~ 16.4 h*	*Sharova et al., 2009; Wang & Liu, 2022*
H. sapiens cell lines	A_T/Ω *(transcription factor concentration)*	$10^{-9} \sim 10^{-7} M$	*Milo et al., 2016*

Regarding the typical Fano factor ranges observed across different cell types, recent experimental studies have primarily reported Fano factor values in mouse embryonic stem cells and Jurkat T

lymphocytes, typically ranging between 1 and 20 [R13]. This range is consistent with our simulation results under biologically plausible conditions (Fig. 3c). Although these data do not yet cover a wide spectrum of cell types, these are the only quantitative measurements we could find. If you are aware of additional references, we would greatly appreciate your suggestions. Now, we have added the reference to the revised Results section as follows:

Page 8 Line 178:

Accordingly, under biologically relevant conditions, only the combination of sequestration, blocking, and displacement exhibits a low Fano factor ranging from 1 to 20—consistent with the experimentally observed range in mammalian cells—and is uniquely capable of reducing it to values close to 1 [37] (Fig. 3c).

4. The mechanistic differences between Figure 4a–c and Figure 2a are not entirely clear. While the main text and captions suggest that these figures depict similar underlying mechanisms, there are apparent inconsistencies between them. For instance, "sequestration" is labeled under the gene-activator complex in Fig. 4a, while "blocking" is labeled under the same complex in Fig. 4b. Moreover, Fig. 4c lacks a gene-activator complex altogether. While the meaning of the solid arrows in these figures is clear, the roles of the dashed arrows and the solid or dashed lines without arrowheads are not immediately obvious. Clarifying the mechanistic distinctions behind these figures and the meanings represented by these graphical elements would greatly aid the reader's understanding.

[Response] We thank the reviewer for carefully pointing out these ambiguities. Figure 2a was intended to present a simplified schematic of the three repression mechanisms, whereas Figure 4a–c depicts their implementation in the context of specific biological oscillators (circadian clock, NF-κB, and p53–Mdm2). However, as the reviewer noted, these figures all depict the same underlying mechanisms—blocking, sequestration, and displacement—but this similarity was not made sufficiently clear in the original version because of inconsistent representations. To address this, we have clarified the graphical

conventions as follows: (1) Solid arrows represent biochemical reactions such as binding/unbinding, transcription, and degradation; (2) Dashed arrows, which were originally intended to denote removal by displacement, have been replaced with solid arrows to emphasize that they also represent biochemical reactions. Instead, removed components are now indicated by dashed outlines. (3) Lines without arrowheads were remnants of incomplete arrows; to reduce confusion, they have been connected to the appropriate arrows. Accordingly, we have revised the figures as follows:

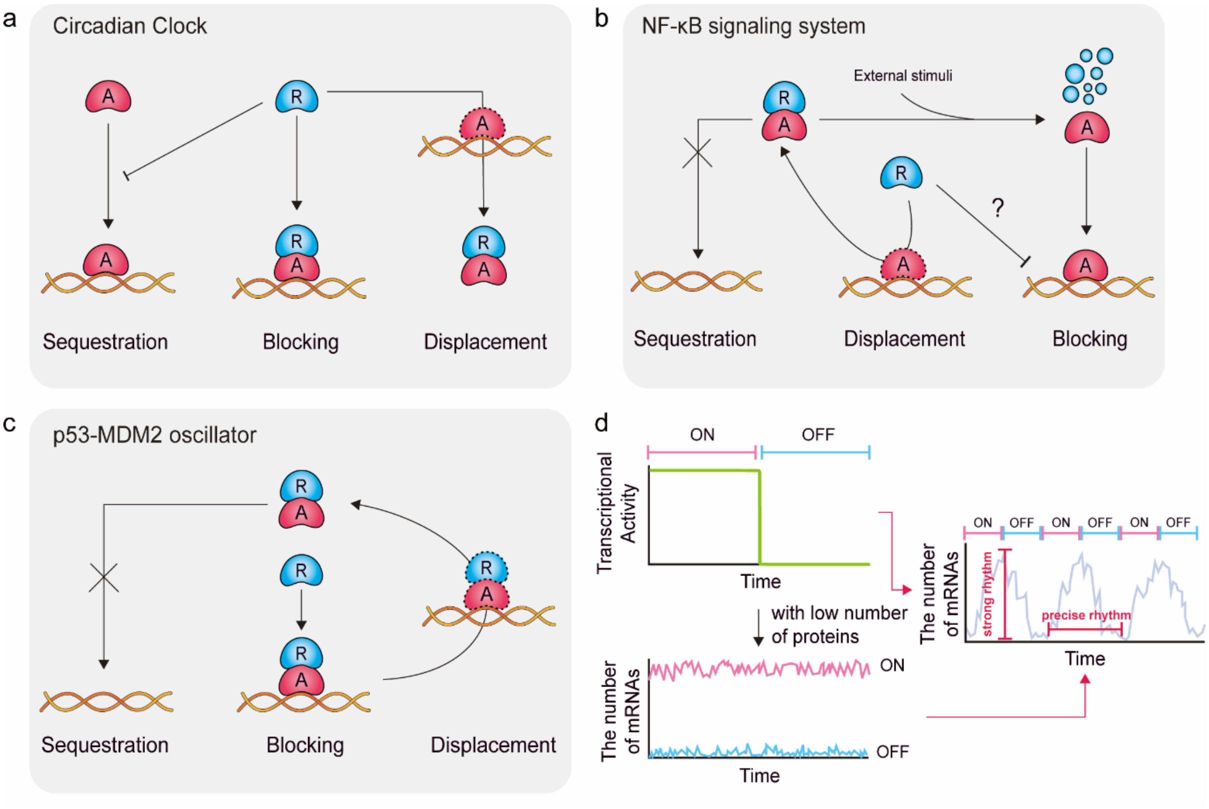

Minor points

1. The authors have computed the transcriptional activities and Fano factors in closed form for several models. Could the authors also comment on whether other quantities—such as the distribution of mRNA counts and the autocorrelation function—can be derived in these models? These quantities provide further insights into stochastic gene expression. For instance: the time-dependent mRNA

distribution has been derived for the model shown in Fig. 1a in:

-Analytical time-dependent distributions for gene expression models with complex promoter switching mechanisms. SIAM Journal on Applied Mathematics, 83(4):1572–1602, 2023,

and the autocorrelation function has been derived for a minimal negative feedback loop in:

-Exact power spectrum in a minimal hybrid model of stochastic gene expression oscillations. SIAM Journal on Applied Mathematics, 84(3):1204–1226, 2024.

A brief comment on the feasibility of similar calculations in the authors' framework would be valuable.

[Response] We sincerely thank the reviewer for raising this important point and for pointing us to these valuable references. We fully agree that additional quantities—such as the full distribution of mRNA counts and the autocorrelation functions—can provide deeper insight into stochastic gene expression beyond the transcriptional activity and Fano factor. As the reviewer noted, these quantities can indeed be derived by applying established analytical methodologies. Specifically, for the multiple-DNA-binding-site model with cooperative binding (Fig. 1a), the full probability distribution can be obtained by applying generating function techniques and simplifying the underlying Markov chains, as demonstrated in recent studies [*SIAM J. Appl. Math.*, 83(4):1572–1602, 2023]. Similarly, the autocorrelation function characterizing noisy rhythmic properties in oscillatory systems (Fig. 4e–g) can be analyzed using linear noise approximation [*SIAM J. Appl. Math.*, 84(3):1204–1226, 2024]. We have now added a paragraph to the Discussion section to acknowledge and discuss these possible analytical extensions, as follows:

Page 14 Line 316:

Finally, we derived analytical expressions for the mean (i.e., transcriptional activity) and the Fano factor of mRNA across different repression models. While our analysis focused on these stationary statistics, recent analytical advances suggest that more detailed stochastic quantities can also be obtained. Specifically, by applying multiscale simplification techniques [71], the full probability distribution of mRNA for the multi–binding-site model (Fig. 1a) could in principle be derived. Similarly, the autocorrelation function of the oscillator model, which we used to characterize noisy rhythmic

properties in biological oscillators (Fig. 4), could be derived using linear noise approximation [72]. Extending our framework in these directions would deepen understanding of the stochastic underpinnings of gene regulation and provide additional validation for the theoretical conclusions drawn here.

2. In the model that combines indirect repression with only sequestration, the following reactions may be missing in Table 1: "R+A \rightarrow R_A" and "R_A \rightarrow R+A".

[Response] We appreciate the reviewer's careful reading. The reactions $R + A \to R_A$ and $R_A \to R + A$ were not explicitly listed in Table 1, because we have assumed that the binding/unbinding reactions between proteins (i.e., R and A) equilibrate much faster than those between proteins and DNA (i.e., A and E_F, R and E_A, and R_A and E_F). As the number of proteins are typically much larger than gene copy number, this assumption is biologically reasonable. Under this assumption, the numbers of proteins (i.e., R, A, and R_A) equilibrate rapidly and can be approximated by their quasi-steady-state approximations (QSSAs) (i.e., the square-root equations shown in the caption of Table 1), rather than by explicitly incorporating reactions between them. To validate this assumption, we compared the current triple-mechanism model (the reduced model; Table 1) with one that explicitly incorporates the binding/unbinding reactions between R and A (a full model) while varying the molar ratio $\widetilde{R_T}$. These simulations confirmed that the current parameter set fully satisfies the conditions required for the QSSA (New Supplementary Figure 2). Specifically, the mRNA trajectories and stationary distributions simulated with the two models are consistent across a range of $\widetilde{R_T} = 0.2,\ 1,$ and 5, supporting the validity of the QSSA assumption. The corresponding results have been added to the Supplementary Information as follows:

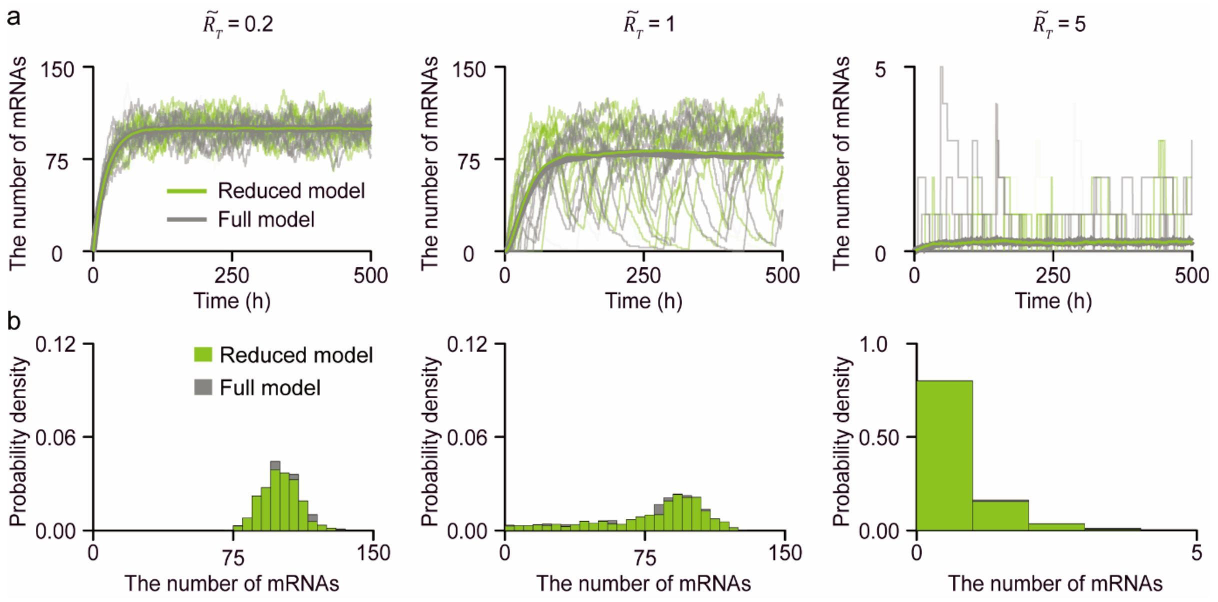

Supplementary Figure 2. The original parameter set does not fully satisfy the conditions required for the quasi–steady-state approximation (QSSA).

(a) Ten representative time-series of mRNA copy numbers (thin lines) and their mean trajectories (thick lines) were obtained from 1,000 stochastic simulations using the model that combines sequestration, blocking, and displacement. The reduced model, in which the numbers of activators and repressors are approximated by their QSSA (gray lines; see Methods), was compared with the full model that explicitly models the binding and unbinding reactions between them (green lines). Simulations were performed while varying the molar ratio $\widetilde{R_T} = 0.2$ *(left),* 1 *(middle), and* 5 *(right), with the original parameter set in Table 1. (b) Probability density functions of the simulated mRNA copy numbers at 500 h, obtained from 1,000 stochastic simulation runs varying the molar ratio* $\widetilde{R_T} = 0.2$ *(left),* 1 *(middle), and* 5 *(right). The full and reduced models show consistent dynamics at each* $\widetilde{R_T}$.

Page 40 Line 747:

Here, n_X is the number of X; A_T, R_T, and D_T are the total number of activators, repressors, and additional target genes, respectively. *Under the assumption that the binding/unbinding reactions between proteins (i.e.,* R *and* A*) equilibrate much faster than those between proteins and DNA (i.e.,* A *and* E_F*,* R *and* E_A*, and* R_A *and* E_F*), the number of proteins (i.e.,* R*,* A*, and* R_A*) equilibrate*

rapidly and can be approximated by their quasi-steady-state approximations (QSSAs). These are given by:

$A(R_T, A_T, K_s) = \frac{A_T - R_T - \Omega K_s + \sqrt{(A_T - R_T - \Omega K_s)^2 + 4\Omega A_T K_s}}{2}$, $R(R_T, A_T, K_s) = \frac{R_T - A_T - \Omega K_s + \sqrt{(A_T - R_T - \Omega K_s)^2 + 4\Omega A_T K_s}}{2}$, and $R_A(R_T, A_T, K_s) = \frac{A_T + R_T + \Omega K_s - \sqrt{(A_T - R_T - \Omega K_s)^2 + 4\Omega A_T K_s}}{2}$. *This QSSA assumption has been validated through stochastic simulations (Supplementary Fig. 2).*

3. Lines 156–158 are somewhat ambiguous in placement. It is unclear whether these lines are part of the caption for Table 1, the caption for Figure 1, or part of the main text. Please consider revising for clarity.

[Response] We appreciate your feedback regarding lines 156-158. We totally agree that their placement may interrupt the reader's reading flow. To improve readability and ensure better presentation, we have relocated all figures and tables to the end of the manuscript.

4. Line 174: "E_A" should likely be "E_R".

[Response] We thank the reviewer for catching this mistake. We have corrected "E_A" to "E_R" in Line 174 as follows:

Page 7 Line 146: Additionally, R *removes* A *from* E_R with the form of R_A at a rate k_d, whereas R_A can bind to DNA at a rate k_f reversely (displacement; Fig. 2a, top right, and Table 1).

REFERENCES

R1. Milo R, & Phillips, R. Cell Biology by the numbers2015.

R2. Sharova LV, Sharov AA, Nedorezov T, Piao Y, Shaik N, Ko MS. Database for mRNA half-life of 19 977 genes obtained by DNA microarray analysis of pluripotent and differentiating mouse

R3. Wang C, Liu H. Factors influencing degradation kinetics of mRNAs and half-lives of microRNAs, circRNAs, lncRNAs in blood in vitro using quantitative PCR. Sci Rep. 2022;12(1):7259. Epub 20220504. doi: 10.1038/s41598-022-11339-w. PubMed PMID: 35508612; PubMed Central PMCID: PMCPMC9068688.

R4. Williamson MP. Protein Binding: A Fuzzy Concept. Life (Basel). 2023;13(4). Epub 20230323. doi: 10.3390/life13040855. PubMed PMID: 37109384; PubMed Central PMCID: PMCPMC10145316.

R5. Zou R, Zhou Y, Wang Y, Kuang G, Agren H, Wu J, et al. Free Energy Profile and Kinetics of Coupled Folding and Binding of the Intrinsically Disordered Protein p53 with MDM2. J Chem Inf Model. 2020;60(3):1551–8. Epub 20200226. doi: 10.1021/acs.jcim.9b00920. PubMed PMID: 32053358.

R6. Bergqvist S, Alverdi V, Mengel B, Hoffmann A, Ghosh G, Komives EA. Kinetic enhancement of NF-kappaBxDNA dissociation by IkappaBalpha. Proc Natl Acad Sci U S A. 2009;106(46):19328–33. Epub 2009/11/06. doi: 10.1073/pnas.0908797106. PubMed PMID: 19887633; PubMed Central PMCID: PMCPMC2780789.

R7. Janssens S, Tschopp J. Signals from within: the DNA-damage-induced NF-kappaB response. Cell Death Differ. 2006;13(5):773–84. doi: 10.1038/sj.cdd.4401843. PubMed PMID: 16410802.

R8. Geva-Zatorsky N, Rosenfeld N, Itzkovitz S, Milo R, Sigal A, Dekel E, et al. Oscillations and variability in the p53 system. Mol Syst Biol. 2006;2:2006 0033. Epub 20060613. doi: 10.1038/msb4100068. PubMed PMID: 16773083; PubMed Central PMCID: PMCPMC1681500.

R9. Guo X, Adelaja A, Singh A, Wollman R, Hoffmann A. Modeling heterogeneous signaling dynamics of macrophages reveals principles of information transmission in stimulus responses. Nat Commun. 2025;16(1):5986. Epub 20250701. doi: 10.1038/s41467-025-60901-3. PubMed PMID: 40593748; PubMed Central PMCID: PMCPMC12218168.

R10. Bolognesi B, Lehner B. Reaching the limit. Elife. 2018;7. Epub 20180810. doi: 10.7554/eLife.39804. PubMed PMID: 30095407; PubMed Central PMCID: PMCPMC6086656.

R11. Dekel E, Alon U. Optimality and evolutionary tuning of the expression level of a protein. Nature. 2005;436(7050):588–92. doi: 10.1038/nature03842. PubMed PMID: 16049495.

R12. Frei T, Cella F, Tedeschi F, Gutierrez J, Stan GB, Khammash M, et al. Characterization and mitigation of gene expression burden in mammalian cells. Nat Commun. 2020;11(1):4641. Epub 20200915. doi: 10.1038/s41467-020-18392-x. PubMed PMID: 32934213; PubMed Central PMCID: PMCPMC7492461.

R13. Khetan N, Zuckerman B, Calia GP, Chen X, Garcia Arceo X, Weinberger LS. Single-cell RNA sequencing algorithms underestimate changes in transcriptional noise compared to single-molecule RNA imaging. Cell Rep Methods. 2024;4(12):100933. Epub 20241210. doi: 10.1016/j.crmeth.2024.100933. PubMed PMID: 39662473; PubMed Central PMCID: PMCPMC11704610.

R14. Jia C, Li YM. Analytical Time-Dependent Distributions for Gene Expression Models with Complex Promoter Switching Mechanisms. Siam J Appl Math. 2023;83(4):1572–602. doi: 10.1137/22m147219x. PubMed PMID: WOS:001158083200001.

R15. Jia C, Qian H, Zhang MQ. Exact Power Spectrum in a Minimal Hybrid Model of Stochastic Gene Expression Oscillations. Siam J Appl Math. 2024;84(3):1204–26. doi: 10.1137/23m1560914. PubMed PMID: WOS:001301427500018.
